# An early endothelial cell–specific requirement for Glut1 is revealed in Glut1 deficiency syndrome model mice

**DOI:** 10.1172/jci.insight.145789

**Published:** 2021-02-08

**Authors:** Maoxue Tang, Sarah H. Park, Sabrina Petri, Hang Yu, Carlos B. Rueda, E. Dale Abel, Carla Y. Kim, Elizabeth M.C. Hillman, Fanghua Li, Yeojin Lee, Lei Ding, Smitha Jagadish, Wayne N. Frankel, Darryl C. De Vivo, Umrao R. Monani

**Affiliations:** 1 Department of Neurology and; 2 Center for Motor Neuron Biology and Disease, Columbia University Irving Medical Center, New York, New York, USA.; 3 Department of Genetics & Development and the Institute for Genomic Medicine, Columbia University, New York, New York, USA.; 4 Departments of Biomedical Engineering and Radiology, Mortimer B. Zuckerman Mind Brain Behavior Institute and Kavli Institute for Brain Science, Columbia University, New York, New York, USA.; 5 Fraternal Order of Eagles Diabetes Research Center and Division of Endocrinology and Metabolism, Carver College of Medicine, University of Iowa, Iowa City, Iowa, USA.; 6 Columbia Stem Cell Initiative and Department of Microbiology & Immunology, Columbia University Irving Medical Center, New York, New York, USA.; 7 Rare & Neurological Diseases Research, Sanofi Genzyme, Framingham, Massachusetts, USA.; 8 Department of Pathology & Cell Biology, Columbia University Irving Medical Center, New York, New York, USA.

**Keywords:** Neuroscience, Monogenic diseases, Mouse models, Neurological disorders

## Abstract

Paucity of the glucose transporter-1 (Glut1) protein resulting from haploinsufficiency of the *SLC2A1* gene arrests cerebral angiogenesis and disrupts brain function to cause Glut1 deficiency syndrome (Glut1 DS). Restoring Glut1 to Glut1 DS model mice prevents disease, but the precise cellular sites of action of the transporter, its temporal requirements, and the mechanisms linking scarcity of the protein to brain cell dysfunction remain poorly understood. Here, we show that Glut1 functions in a cell-autonomous manner in the cerebral microvasculature to affect endothelial tip cells and, thus, brain angiogenesis. Moreover, brain endothelial cell–specific Glut1 depletion not only triggers a severe neuroinflammatory response in the Glut1 DS brain, but also reduces levels of brain-derived neurotrophic factor (BDNF) and causes overt disease. Reduced BDNF correlated with fewer neurons in the Glut1 DS brain. Controlled depletion of the protein demonstrated that brain pathology and disease severity was greatest when Glut1 scarcity was induced neonatally, during brain angiogenesis. Reducing Glut1 at later stages had mild or little effect. Our results suggest that targeting brain endothelial cells during early development is important to ensure proper brain angiogenesis, prevent neuroinflammation, maintain BDNF levels, and preserve neuron numbers. This requirement will be essential for any disease-modifying therapeutic strategy for Glut1 DS.

## Graphical abstract

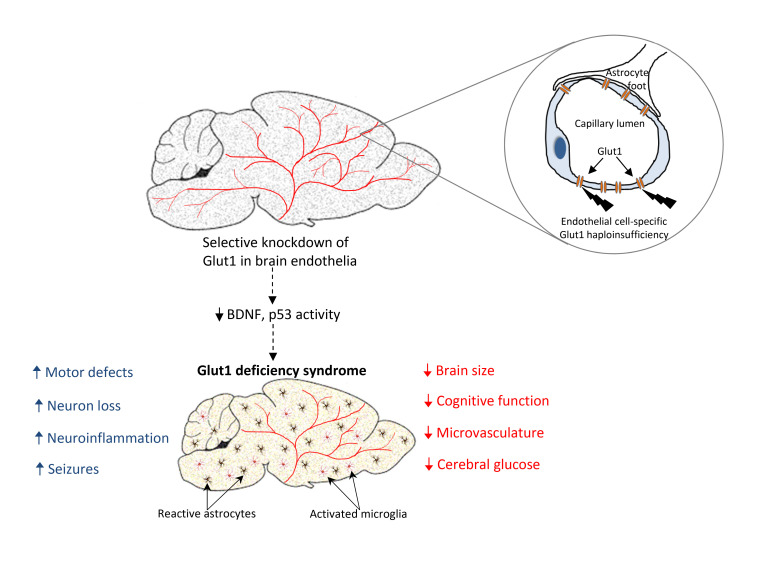

## Introduction

Heterozygous mutations in the *SLC2A1* gene and reduced levels of its translated product, the glucose transporter-1 (Glut1) protein, elicit neuroglycopenia in what is known as Glut1 deficiency syndrome (Glut1 DS; ref. 1). The classic Glut1 DS phenotype is defined by intractable, infantile-onset seizures, cognitive dysfunction, acquired microcephaly (decelerating head growth), and a complex movement disorder combining features of spasticity, ataxia, and dystonia (2, 3). An expanded Glut1 DS phenotype comprising hemolytic anemias and exercise-induced dyskinesia has more recently been described (4). The expanded phenotype, combined with reports that *SLC2A1* mutations account for approximately 1% of idiopathic generalized epilepsies (5) and approximately 10% of absence epilepsies (6), suggests that the disease is more prevalent than originally thought; it is estimated that there are at least 4500 Glut1 DS patients in the US alone (7).

The Glut1 protein is widely expressed but is especially abundant in brain endothelial cells (ECs) and astrocytes (8, 9), where it transports glucose across the blood-brain barrier (BBB) and into the cerebral parenchyma to fuel the high energy requirements of brain neurons. Low Glut1 disrupts this supply and is reflected in greatly reduced glucose concentrations in cerebrospinal fluid (CSF) — a condition referred to as hypoglycorrhachia (10). Although the molecular cause of Glut1 DS was described more than 2 decades ago, there is still no truly effective disease-modifying treatment for the disorder. The current standard of care primarily involves treating patients with a high-fat ketogenic diet. While the diet does mitigate seizure activity, it does not have a measurable effect on other disease symptoms (11).

While investigating links between reduced Glut1 resulting from *SLC2A1* gene haploinsufficiency and brain dysfunction in Glut1 DS, we found that paucity of the transporter arrests brain angiogenesis (12). Development of the brain microvasculature was preserved — and Glut1 DS averted — by systemic administration of an AAV9-Glut1 construct into model mice. While these results highlighted 1 promising therapeutic strategy for Glut1 DS, they did not identify the precise cellular sites of action of the Glut1 protein or define the consequences of a potential loss over time of Glut1-transduced cells such as brain endothelia; little light was cast on Glut1 effectors that might account for selective brain dysfunction in Glut1 DS. Here, we show that brain ECs constitute a critical cellular site of action of the Glut1 protein. Selectively inducing haploinsufficiency to deplete Glut1 in these cells evoked all the major phenotypic characteristics of Glut1 DS. Moreover, we discovered that Glut1 haploinsufficiency has a particularly devastating effect on a subtype of ECs known as tip cells; tip cells are critical to brain microvasculature expansion (13, 14). Depletion of Glut1 in ECs resulted in fewer and less elaborate tip cells. Such depletion also resulted in lower levels of cerebral brain-derived neurotrophic factor (BDNF), an important determinant of neuronal survival, differentiation, and plasticity and one likely effector of the decidedly neurological Glut1 DS phenotype. Indeed, reduced BDNF was found to correlate with fewer brain neurons in Glut1 DS mutants.

Controlled systemic depletion of Glut1 at various postnatal time points demonstrated that inducing haploinsufficiency is most deleterious in young mice when the brain microvasculature is actively expanding. In fact, inactivating 1 copy of the *Slc2a1* gene in P2 mice was as injurious to the health of the brain and the organism at large as a constitutively haploinsufficient state. Depleting the protein at P14 was less damaging, whereas inducing the haploinsufficient state during adulthood caused minimal disease — despite marked hypoglycorrhachia. We conclude that targeting brain endothelia — and, specifically, endothelial tip cells — will be important in preventing the cerebral microvasculature defects characteristic of Glut1 DS and developing an effective treatment for the human disease. Considering the period of postnatal development when brain angiogenesis is most active, our results also imply that early treatment will likely be more effective than delayed interventions. Finally, our observations suggest that such treatments need not be chronic. A brief period of treatment administered early to ensure normal development of the brain microvasculature and neuronal circuitry may provide life-long protection against the disease-causing effects of Glut1 paucity.

## Results

### An EC-autonomous effect of Glut1 in triggering the Glut1 DS phenotype.

Glut1 is the major cerebral glucose transporter and is expressed at particularly high levels in ECs that line brain capillaries (9). Systemic Glut1 haploinsufficiency results in fewer brain capillaries and triggers Glut1 DS (12). To determine if this is a cell-autonomous effect of Glut1 in ECs, we selectively depleted the transporter in these cells. To do so, we generated double-transgenic mice (*Tie2-Cre;Glut1^fl/+^*) harboring a floxed *Slc2a1* (*Glut1*) allele and an EC-specific (*Tie2*) Cre driver (15, 16). The approach avoided completely ablating Glut1 in endothelia and, in contrast to a recent report (17), preserved the haploinsufficient state of human Glut1 DS.

We began by ensuring that systemically engineered haploinsufficiency in *Glut1^fl/+^* mice triggered a Glut1 DS phenotype. This was accomplished by breeding the mice to animals carrying a Zp3-Cre transgene that inactivates the floxed allele in the female germline (18). *Glut1*^Δ/+^** progeny of *Zp3-Cre;Glut1^fl/+^* were then subject to a battery of previously established outcome measures to ensure that *Glut1*^Δ^ is a functionally null allele and that mutants heterozygous for it represent a bona fide model of Glut1 DS. We found that 5-month-old *Glut1*^Δ/+^** mutants do indeed exhibit a very similar disease phenotype to that observed in *Glut1^+/–^* mice carrying a constitutive KO allele (19). Glut1 levels in brain tissue and concentrations of CSF glucose in the *Glut1*^Δ/+^** mutants were reduced, micrencephaly induced, and motor performance impaired (Supplemental Figure 1, A–F; supplemental material available online with this article; https://doi.org/10.1172/jci.insight.145789DS1). We also saw an increase in the frequency of spike-wave discharges (SWDs), the electrographic correlate of absence seizures, a common form of generalized epilepsy previously noted in *Glut1^+/–^* mice (Supplemental Figure 1G; ref. 19). Consistent with these defects, we found reduced numbers of brain capillaries in mutants — a sign of impaired brain angiogenesis (Supplemental Figure 2). The overall Glut1 DS phenotype in *Glut1*^Δ/+^** mutants suggested that mice carrying the inducible floxed *Glut1* allele would be useful in investigating the tissue-specific and temporal requirements for the Glut1 protein.

**Figure 1:**
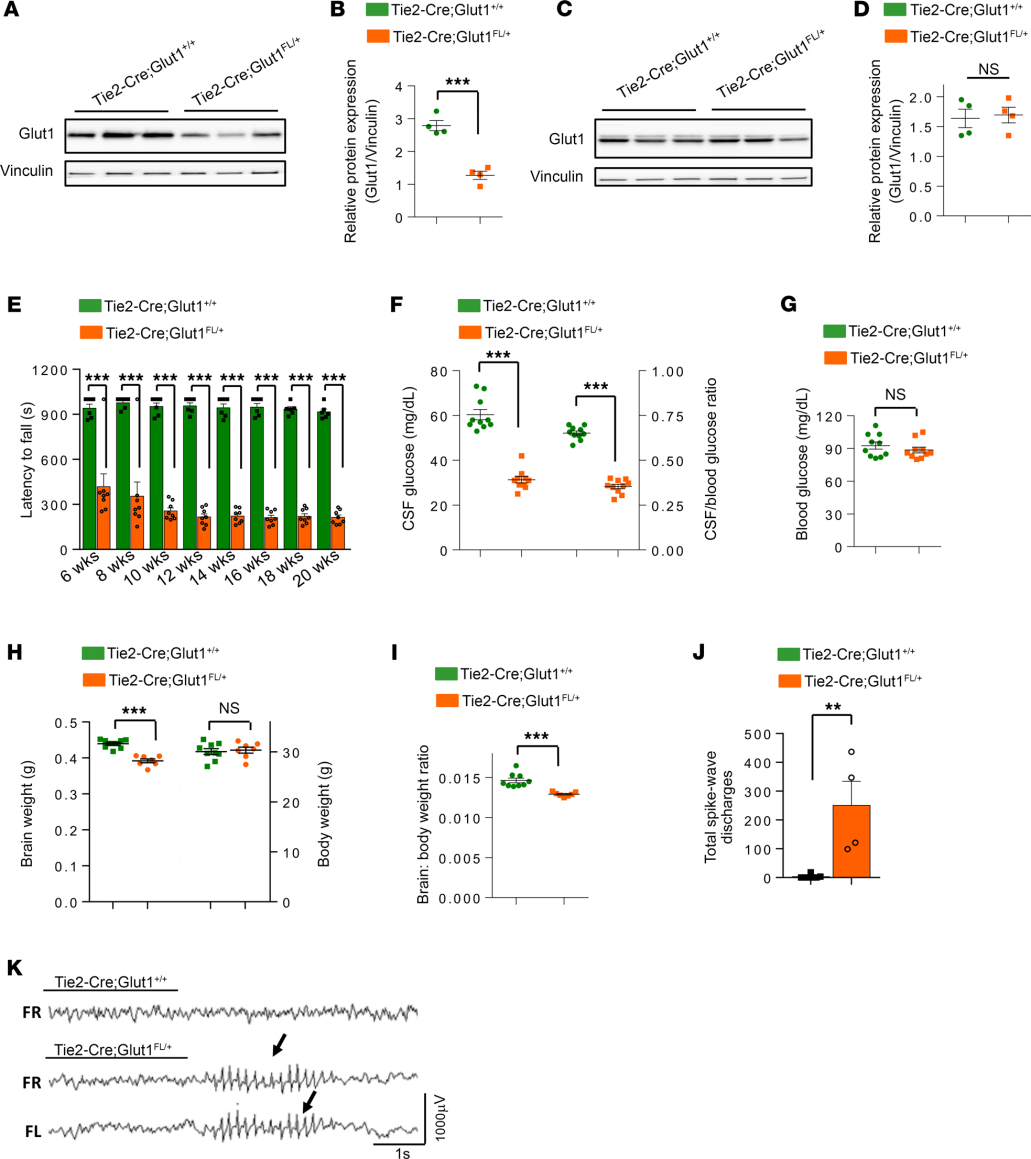
Selective Glut1 haploinsufficiency in endothelial cells (ECs) is sufficient to cause disease. (**A** and **B**) Western blot (**A**) and image analysis of the blot (**B**) provides evidence of reduced Glut1 in brain capillary fractions of 5-month-old *Tie2-Cre;Glut1^fl/+^* mutants. (**C**) Western blot showing equivalent levels of Glut1 in brain neuropil fractions of *Tie2-Cre;Glut1^fl/+^* mutants and controls. (**D**) Quantified results of Glut1 levels in **C**. ***P < 0.001, *t* test, *n* = 4 mice for results in **B** and **D**. (**E**) Reduced time on the rotarod is indicative of motor dysfunction in *Tie2-Cre;Glut1^fl/+^* mutants; ***P < 0.001, *t* test, *n* ≥ 6 mice of each cohort. (**F** and **G**) Reduced CSF glucose and CSF/blood glucose ratios (**F**) despite normal blood glucose concentrations in 5-month old *Tie2-Cre;Glut1^fl/+^* mutants (**G**). (**H** and **I**) Brain size as assessed by the weight of the organ and brain/body weight ratios is abnormally low in the mutants (**H** and **I**), whereas body weight is unchanged (**H**). ***P < 0.001, *t* test, *n* = 10 mice for results in **F**–**I**. (**J**) Increased spike-wave discharges (SWDs) are evidence of EEG abnormalities in *Tie2-Cre;Glut1^fl/+^* mutants; **P < 0.01, Mann-Whitney *U* test, *n* = 4 mice. Mice were analyzed over a 48-hour period. (**K**) Representative EEG traces in mutant and control mice showing SWDs (arrows) in the former.

We next tested the effects of selectively depleting Glut1 in ECs. Accordingly, *Tie2-Cre;Glut1^fl/+^* mice were subject to the same assays utilized in analyzing the *Glut1*^Δ/+^** mutants. We first ensured selective knockdown of Glut1 in the brain microvasculature. Brain tissue was separated into fractions enriched for blood vessels on the one hand and the parenchyma (neurons and glia) on the other. Glut1 protein levels were then estimated. As expected, whereas brain capillaries of *Tie2-Cre;Glut1^fl/+^* expressed significantly lower levels of Glut1, brain fractions comprising nervous tissue did not (Figure 1, A–D). An assessment of motor function on the rotarod demonstrated that selectively depleting Glut1 in ECs significantly impaired locomotion between 6 weeks and 20 weeks of age (Figure 1E). This was accompanied by marked hypoglycorrhachia and correspondingly low CSF/blood glucose ratios (Figure 1F). Consistent with earlier findings in model mice and patients (2, 19), blood glucose levels in the mutants remained unchanged, brain size and brain/body weight ratios were reduced, and SWD frequency increased (Figure 1, G–K). Considering the overall pathology we observed in the *Tie2-Cre;Glut1^fl/+^* mutants, we conclude that selectively inducing *Glut1* haploinsufficiency in ECs is sufficient to trigger a Glut1 DS disease phenotype. By extension, we reason that brain endothelia constitute a critical cellular site of action of Glut1.

### An EC-autonomous effect of Glut1 in regulating brain angiogenesis.

**Figure 2:**
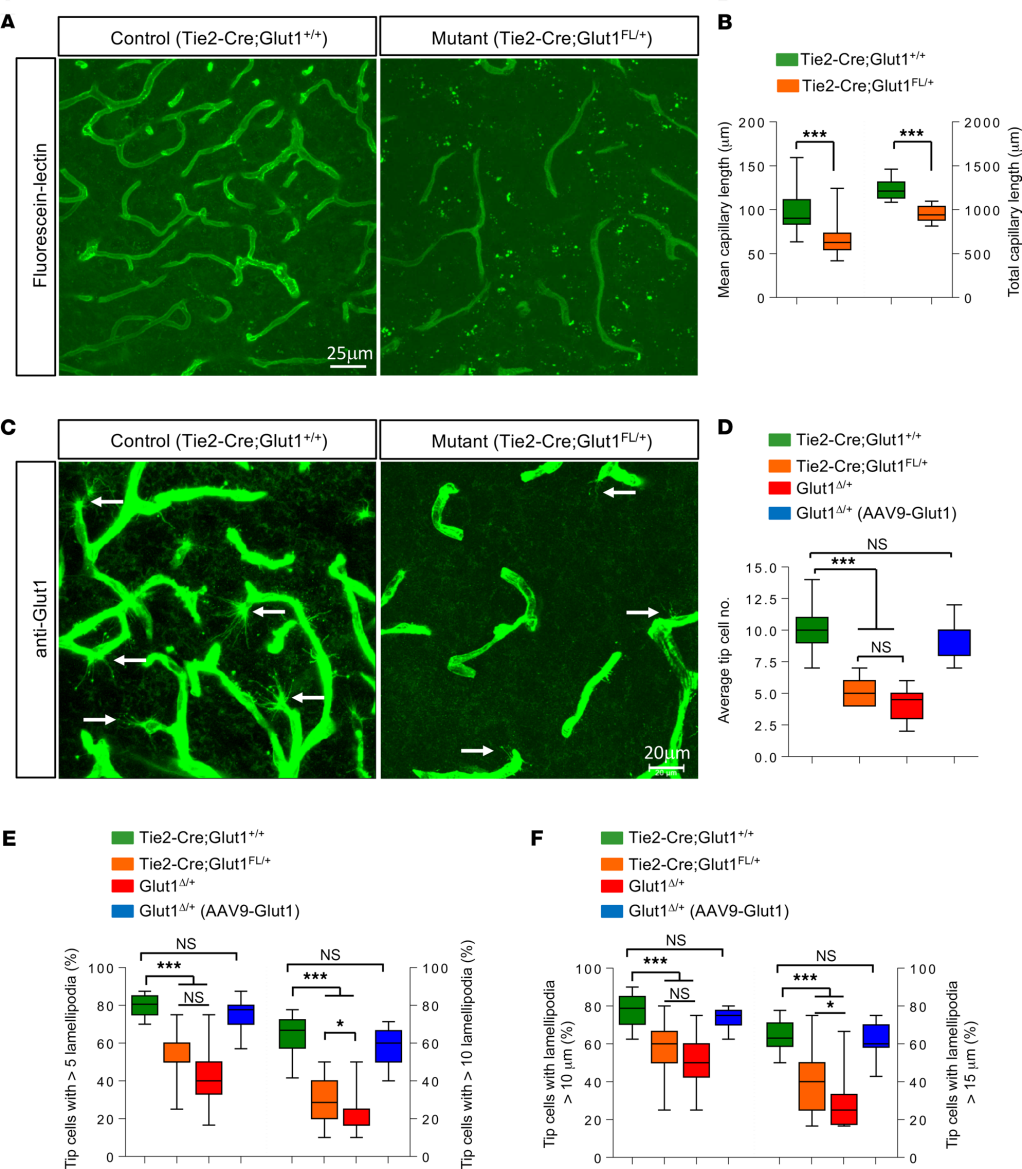
Selective depletion of Glut1 in ECs arrests brain angiogenesis and causes tip cell defects. (**A**) Thalamic sections of *Tie2-Cre;Glut1^fl/+^* mutants and littermate controls stained with labeled lectin at 20 weeks of life reveal cerebral angiogenesis defects and diminution of the mutant microvasculature. (**B**) Graph quantifies average and aggregate cerebral capillary length in the mice; ***P < 0.001, *t* test, *n* = 9 regions from each of *n* = 3 mice of each genotype examined. (**C**) Thalamic sections of *Tie2-Cre;Glut1^fl/+^* mutants and littermate controls stained at P14 for Glut1 reveals fewer tip cells (arrows) in mutants. (**D**) Quantified numbers of tip cells in mutants and relevant controls. (**E** and **F**) Tip cells in mutant brains have fewer (**E**) and shorter (**F**) lamellipodia than those of healthy controls or mutants restored for Glut1. *P < 0.05,***P < 0.001, 1-way ANOVA, *n* ≥ 9 regions from each of *n* = 3 mice of each genotype examined for **D**–**F**.

Perhaps the most striking brain pathology described thus far in Glut1 DS model mice is a diminutive microvasculature (12, 20). To determine if this finding, too, is a cell-autonomous effect of Glut1 in ECs, we quantified the density of the capillary network in the thalamus of *Tie2-Cre;Glut1^fl/+^* mutants, a region of the brain we previously examined (12). We found that selectively depleting Glut1 in ECs was just as damaging to the microvasculature of this region of the brain as an organism-wide paucity of the protein. Brains of 5-month-old mutants probed with fluorescently labeled lectin had approximately 40% fewer vessels than brains of *Tie2-Cre;Glut1^+/+^* controls (Figure 2, A and B). To investigate the cellular basis of this anomaly, we stained brain sections with an antibody against Glut1 and examined the numbers and morphology of endothelial tip cells. These cells pioneer new blood vessels and are easily identified in P14 mice when brain angiogenesis is underway (14). We enumerated only half as many tip cells in *Tie2-Cre;Glut1^fl/+^* mutants at this point as we did in age-matched *Tie2-Cre;Glut1^+/+^* controls. This was true of sections from both thalamus and cortex (Figure 2, C and D, and Supplemental Figure 3, A and B). Moreover, the remaining tip cells in mutant brains stained less intensely, appeared stunted as if arrested in their development, and had significantly fewer and smaller lamellipodia — finger-like processes at the leading edge of tip cells used to sense the brain microenvironment before new vessels sprout (Figure 2, E and F, and Supplemental Figure 3, C and D). Lamellipodia of tip cells in control animals frequently stained especially intensely for Glut1 at their extremities, giving the striking appearance of orbs at the distal ends of the processes (Supplemental Figure 3A). This focal concentration of Glut1 suggests a particularly high requirement for the transporter and, thus, glucose at the “sensing” tips of endothelial lamellipodia. To ensure that defective endothelial tip cells are a genuine attribute of the Glut1 DS brain microvasculature, we examined their morphology in 2 additional cohorts of P14 mice. First, we examined the cells in *Glut1*^Δ/+^** mutants. Second, we examined them in *Glut1*^Δ/+^** mutants restored at P2 for Glut1 by means of an AAV9-Glut1 vector. Congruent with the defects being a direct result of reduced Glut1 in brain endothelia, we found fewer and less elaborate tip cells in untreated *Glut1*^Δ/+^** mutants. In contrast, and consistent with the mitigating effects of AAV9-Glut1 treatment, repletion of the Glut1 protein generally preserved tip cell numbers and ameliorated their defects (Figure 2, D–F, and Supplemental Figure 3, A–D). These results suggest that the diminutive microvasculature of the Glut1 DS brain is a direct consequence of the effects of low Glut1 on brain endothelia in general — and on endothelial tip cells, specifically.

### Controlled depletion of Glut1 in neonatal mice arrests brain angiogenesis and triggers Glut1 DS.

Constitutive Glut1 haploinsufficiency in all tissues or just ECs causes Glut1 DS. Moreover, restoring the transporter to prevent disease is most effective when implemented in the first postnatal week of life (12), suggesting a relatively early requirement for the protein. To directly and unequivocally demonstrate an early requirement for Glut1 and furthermore accurately define when during postnatal life Glut1 is critically needed, we generated a potentially novel line of mice in which haploinsufficiency can be induced in a precisely timed manner. To do so, mice harboring the floxed *Glut1* allele were bred to animals transgenic for a ubiquitously expressed tamoxifen-induced (TM-induced) CreER driver (21). Glut1 haploinsufficiency was induced in double-transgenic *CreER;Glut1^fl/+^* mice by administering TM at P2.

**Figure 3:**
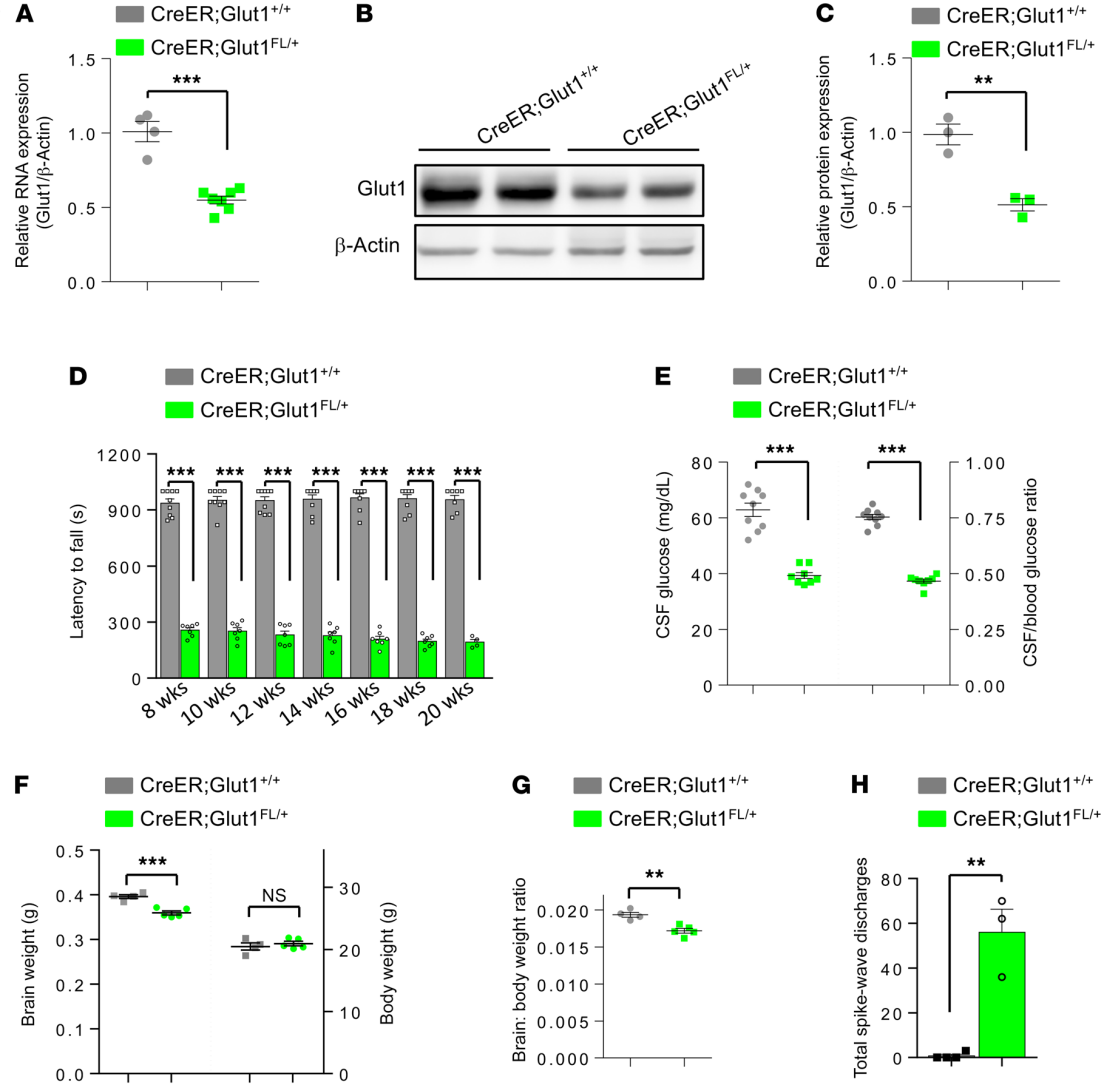
An early postnatal requirement for Glut1 is revealed in model mice. (**A**) Inactivation of the floxed *Glut1* allele at P2 results in a 50% loss of Glut1 transcript in 5-month-old *CreER;Glut1^fl/+^* mice. (**B**) Western blot of brain tissue shows a corresponding loss of protein. (**C**) Quantified result of blot; **P < 0.01, ***P < 0.001, *t* test, *n* = 3 mice analyzed in **A** and **C**. (**D**) Mutants perform poorly on the rotarod at all points selected for analysis; ***P < 0.001, *t* test, *n* ≥ 5 mice tested. (**E**–**H**) Five-month-old mutants also exhibit hypoglycorrhachia (**E**), micrencephaly (**F** and **G**), and increased seizure activity (**H**) in the form of spike-wave discharges in EEG tests. **P < 0.01, ***P < 0.001, *t* test for **E** (9 mice of each genotype), **F**, and **G** (4 controls, 5 mutants); Mann-Whitney *U* test for **H** (3 controls, 4 mutants).

**Figure 4:**
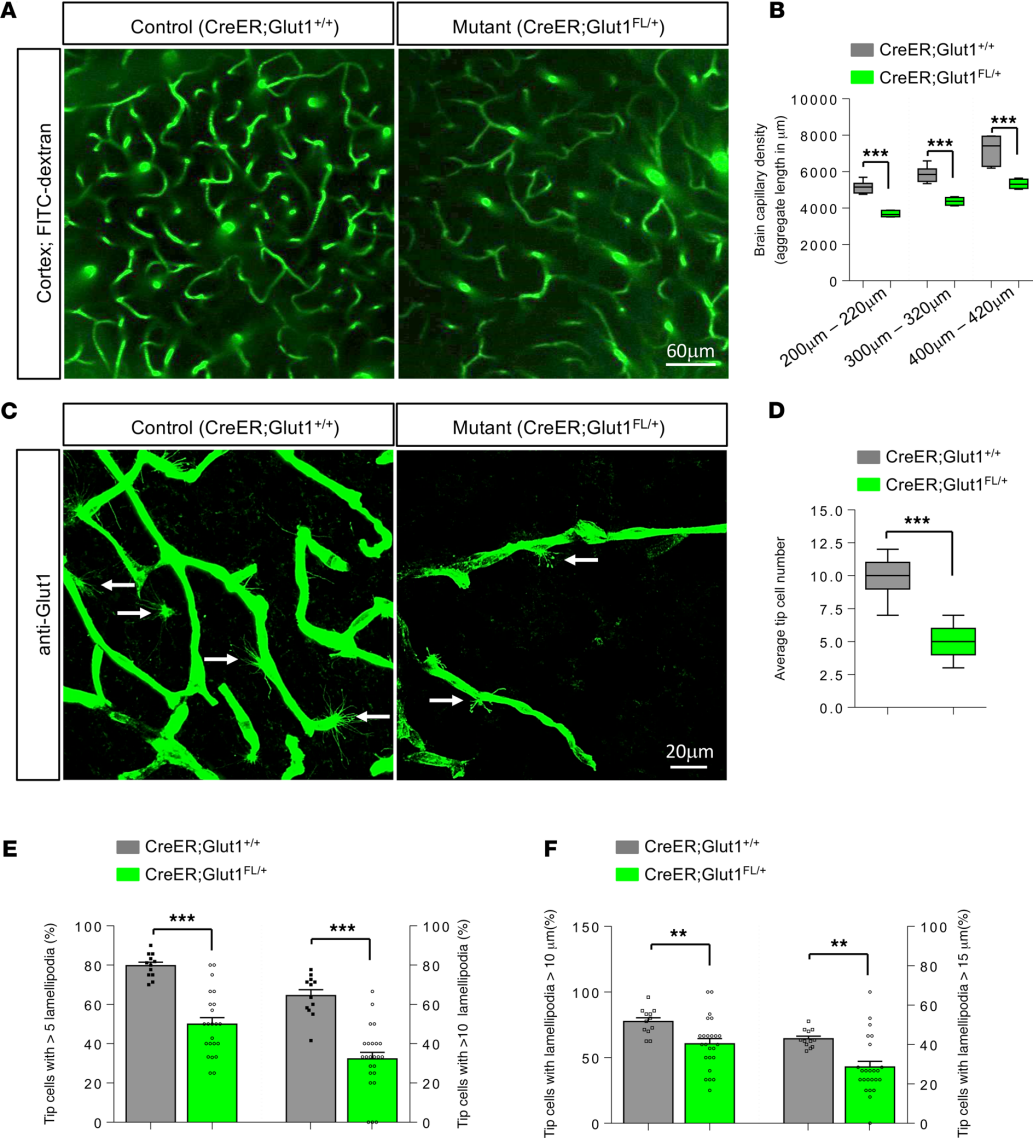
Controlled depletion of Glut1 in early postnatal life triggers brain microvasculature defects. (**A**) Representative live-imaging experimental result of the cortical brain microvasculature, at a depth of 200–220 μm, of a 5-month-old mutant and a littermate control. Note reduced density of capillaries in the mutant. (**B**) Graphical representation of cortical capillary densities at indicated depths in the 2 groups of mice following 2-photon live imaging; ***P < 0.001, *t* test, *n* ≥ 9 regions from each of *n* = 6 mice of each cohort. (**C**) Cortical brain sections of P14 mice stained for Glut1 reveal a relative paucity and defects of mutant endothelial tip cells (arrows). (**D**–**F**) Statistically analyzed results of tip cell numbers (**D**), lamellipodia counts (**E**), and lamellipodia size (**F**) in mutants and controls. ** *P* < 0.01, ***P < 0.001, *t* test, *n* = 9 regions from each of *n* = 3 mice of each genotype examined for **D**–**F**.

*CreER;Glut1^fl/+^* mice administered TM at P2 were visually indistinguishable from similarly treated littermate controls (*CreER;Glut1^+/+^*) despite robust knockdown of both Glut1 transcript and protein (Figure 3, A–C). However, their performance on the rotarod was clearly impaired (Figure 3D) and no better at 20 weeks of age than that of constitutive heterozygotes (latency to fall in seconds, *CreER;Glut1^fl/+^* = 193 ± 14.5 seconds; *Glut1*^Δ/+^**, 185 ± 6.7 seconds; *P* = 0.67, 2-tailed *t* test, *n* = 10 mice of each cohort). Consistent with the poor motor performance, we found greatly reduced levels of CSF glucose (Figure 3E) and significant micrencephaly (Figure 3, F and G) in the induced mutants. Additionally, akin to findings in constitutive *Glut1*^Δ/+^** and *Tie2-Cre;Glut1^fl/+^* mutants, these phenotypes were accompanied by electro-encephalograph (EEG) abnormalities in the form of SWDs (Figure 3H; ref. 18). Examination of the brain microvasculature of the mutants revealed a sparser capillary density at 5 months of age (Figure 4, A and B) and fewer cortical and thalamic endothelial tip cells in P14 juvenile mice (Figure 4, C and D, and Supplemental Figure 4, A and B). Tip cells that were recognizable resembled those in the developing brains of *Glut1*^Δ/+^** and *Tie2-Cre;Glut1^fl/+^* mutants, appearing arrested in development with relatively few, mostly undersized lamellipodia (Figure 4, E and F, and Supplemental Figure 4, C and D).

In spite of the observed pathology and evidence of Glut1 DS in the *CreER;Glut1^fl/+^* depleted of protein at P2, all mutants remained viable. We therefore attempted to reduce Glut1 levels even further to below heterozygous levels to ascertain minimum amounts of the protein required to sustain life. To do so, we effected depletion at P2 in mice harboring 2 copies of the *Glut1* floxed allele (*CreER;Glut1^fl/fl^*). As expected, we found that Glut1 in brain tissue of approximately 3-week-old mice was considerably lower than that in haploinsufficient animals (Supplemental Figure 5, A–C). Transcript levels in brain tissue were assessed at approximately 30% of WT levels; Glut1 protein concentrations were even lower at approximately 15% of WT levels. Significantly, most mutants generated in this manner succumbed to disease before 2 months of age (Supplemental Figure 5D). In aggregate, our results suggest that not only is there an early requirement for Glut1 to prevent onset of disease, but also a minimum amount, between 15% and 50% of WT levels of the protein, to sustain life.

### Early, EC-derived gliosis is a characteristic feature of the Glut1 DS brain.

**Figure 5:**
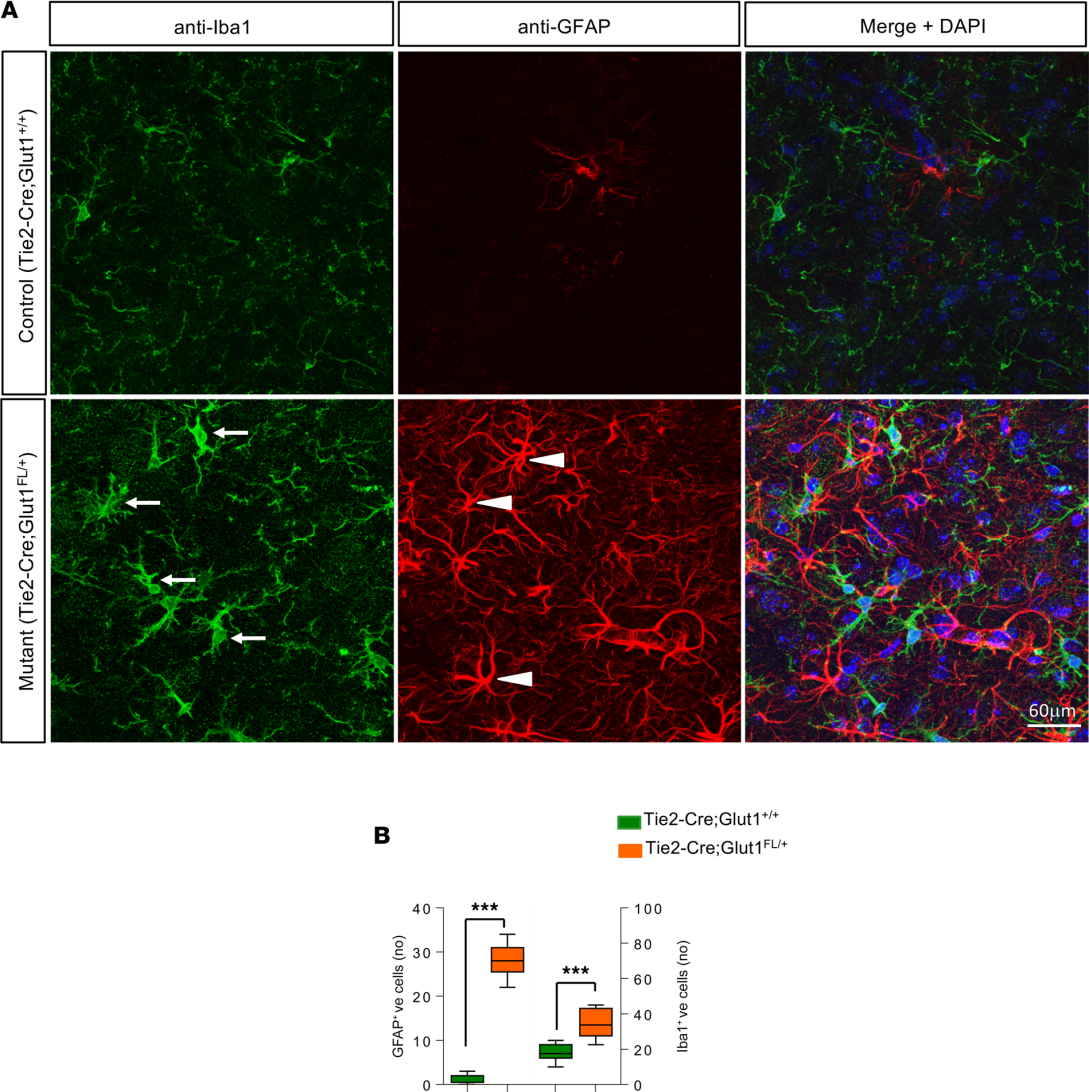
Selective loss of Glut1 in ECs evokes cerebral neuroinflammation. (**A**) Representative thalamic brain sections from 1-month-old *Tie2-Cre;Glut1^fl/+^* mutants and controls stained with antibodies against Iba1 and GFAP. Selective inactivation of even 1 *Glut1* allele in ECs is sufficient to cause a marked increase in activated microglia (arrows) and reactive astrocytes (arrowheads). (**B**) Graph depicting enumeration of activated microglia and reactive astrocytes in thalamic brain from 1-month-old *Tie2-Cre;Glut1^fl/+^* mutants and controls. ***P < 0.001, *t* test, *n* = 9 regions from each of *n* = 3 mice of each genotype examined.

Glut1 is expressed not just as a 55 kDa protein by brain ECs, but also as a differentially glycosylated 45 kDa isoform by brain astrocytes (22, 23). Moreover, there is evidence that brain astrocytes become reactive in constitutive Glut1 DS heterozygous mice (24). We therefore also investigated if early postnatal depletion triggered gliosis. We found that treating *CreER;Glut1^fl/+^* mice at P2 to induce Glut1 haploinsufficiency also caused severe neuroinflammation — as assessed in the adult thalamus. This finding featured not only striking GFAP^+^ reactive astrocytosis, but also a marked increase in hypertrophic, activated microglia (Supplemental Figure 6, A and B). To determine if selective loss of Glut1 in ECs triggers the response in glia, we examined the brain tissue of similarly aged *Tie2-Cre;Glut1^fl/+^* mutants. We found a comparable neuroinflammatory response in these mutants, suggesting that reactive gliosis is, at least in part, a non–cell-autonomous effect of Glut1 haploinsufficiency in ECs (Supplemental Figure 6, A and B). Moreover, the effect was obvious in 1-month-old *Tie2-Cre;Glut1^fl/+^* mutants (Figure 5, A and B) and as early as 1 week of age in mice systemically and constitutively haploinsufficient for Glut1 (Supplemental Figure 6, B and C). Collectively, the data imply that neuroinflammation is an important aspect of the overall pathology of the Glut1 DS brain and can be triggered in a non–cell-autonomous manner by low Glut1 in ECs.

### Glut1 haploinsufficiency is associated with depletion of cerebral BDNF and fewer brain neurons.

**Figure 6:**
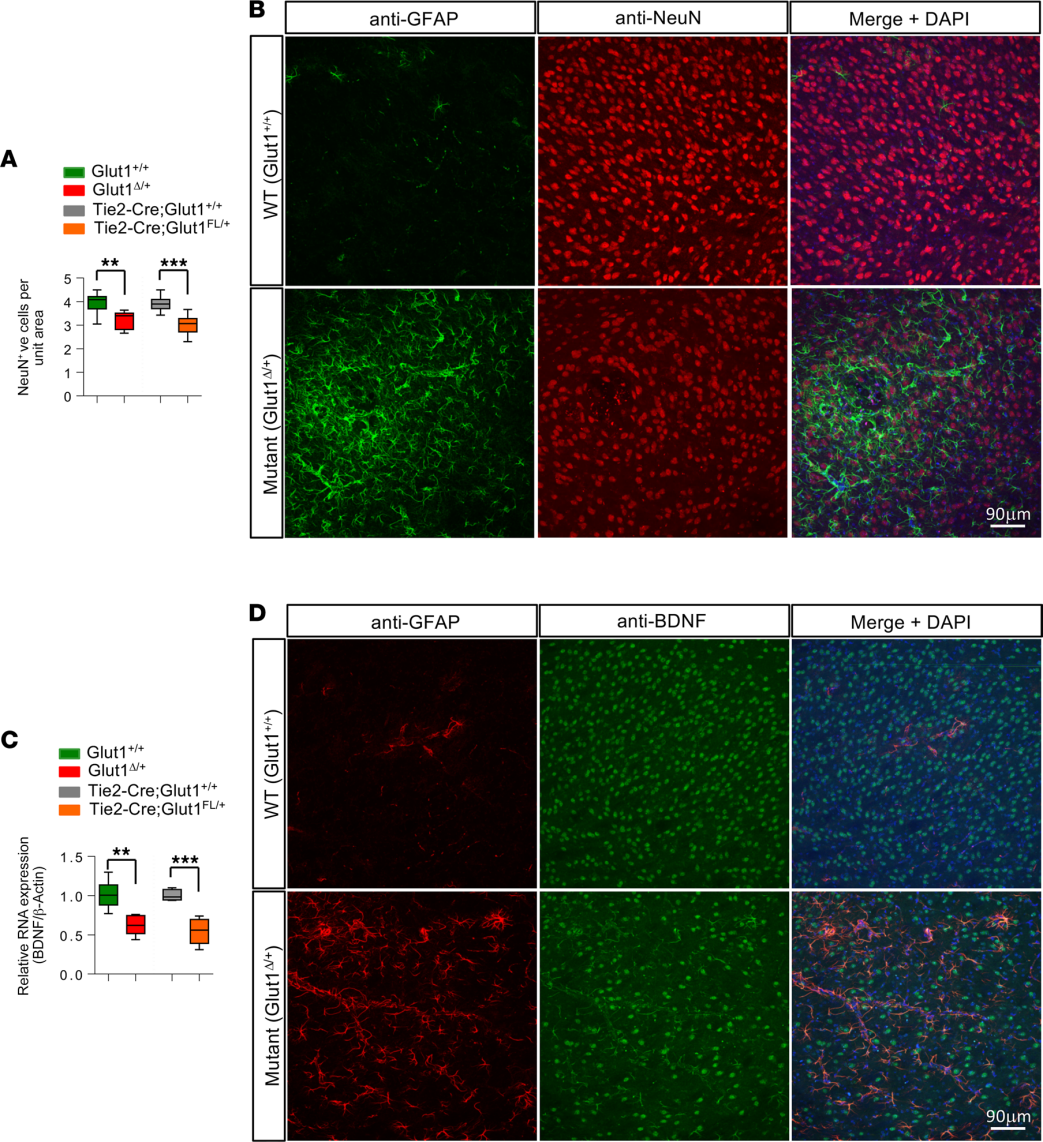
Glut1 haploinsufficiency results in fewer brain neurons and reduced levels of BDNF. (**A**) Enumeration of NeuN^+^ cells in the ventral posteriomedial (VPM) nucleus of the thalamus of mutants either systemically heterozygous (*Glut1^Δ/+^*) or EC-specific haploinsufficient (*Tie2-Cre;Glut1^fl/+^*) for the *Glut1* allele; **P < 0.01, ***P < 0.001, *t* tests, *n* ≥ 3 regions from each of *n* = 3 mice of each genotype examined. (**B**) Representative thalamic sections from *Glut1*^Δ/+^** mutant and control mice dual-stained for GFAP and NeuN to highlight the marked astrocytosis and reduced neurons under conditions of reduced Glut1. (**C**) Quantified results of PCR for BDNF transcripts in brain tissue of mutants either systemically heterozygous (*Glut1*^Δ/+^**) or EC-specific haploinsufficient (*Tie2-Cre;Glut1^fl/+^*) for the *Glut1* allele. **P < 0.01, ***P < 0.001, *t* tests, *n* = 4 mice of each genotype examined. (**D**) Thalamic sections from mutant and control mice dual-stained for GFAP and BDNF illustrate the greater extent of the astrocytosis and relative paucity of BDNF^+^ cells in the former.

Since activated microglia and reactive astrocytosis are frequently associated with neurodegeneration, we next asked if the profound gliosis we had observed in Glut1 haploinsufficient mice might also be associated with neuron loss. Accordingly, we examined the thalami of adult *Glut1*^Δ/+^** and *Tie2-Cre;Glut1^fl/+^* mutants. We discovered that this region of the mutant brain in which gliosis was especially prominent contained approximately 20% fewer neurons in each of the mutants (Figure 6, A and B, and Supplemental Figure 7A). To identify mediators of such loss, we examined *Glut1*^Δ/+^** mutants for cerebral levels of BDNF, a neurotrophin whose expression is reportedly influenced by glycolytic flux and whose effects on cognition via regulation of synaptic plasticity and neuronal survival are well known (25–27). We discovered that BDNF transcripts were only roughly half as abundant in mutant brain (Figure 6C). IHC of mutant brain also revealed fewer BDNF^+^ cells (Figure 6D and Supplemental Figure 7B). To determine if selectively reducing Glut1 in ECs similarly perturbs BDNF levels, we repeated the PCR quantification on brain tissue of *Tie2-Cre;Glut1^fl/+^* mice. Consistent with our earlier assessment of an EC-autonomous disease-causing role for Glut1, we found that brain tissue of *Tie2-Cre;Glut1^fl/+^* mutants also expressed significantly reduced levels of BDNF (Figure 6C).

Additional factors that have recently been implicated in mediating the effects of low Glut1 include the cellular energy sensor, AMP-activated protein kinase (AMPK) and its downstream targets, p53 and p21 (17). Since a role for these factors was revealed under conditions of complete Glut1 ablation, a situation that does not accurately represent the Glut1 DS haploinsufficient state, we reexamined potential alterations in their levels in brain tissue from our heterozygous *Glut1*^Δ/+^** mutants. In contrast to the findings of Veys et al. (17), we did not detect a significant increase in phospho-AMPK protein in the *Glut1*^Δ/+^** mutants (Supplemental Figure 8, A and B). Quantification of p53 transcripts also failed to reveal any change in mutant brain (Supplemental Figure 8C). However, since p53 is activated posttranscriptionally, quantification of its transcripts may not be instructive. Accordingly, we examined mRNA levels of genes commonly induced by activated p53. We found that transcripts of the 5 p53 target genes investigated were reduced, suggesting that while p53 may be an effector of low Glut1, its effects, under conditions of Glut1 haploinsufficiency, are mediated by repressing rather than activating downstream targets (Supplemental Figure 8D). In spite of our inability to detect AMPK or p53 activation, to our knowledge, the altered BDNF observed in our Glut1-deficient mutants provides the first cellular and molecular link between the widely expressed Glut1 protein and the distinct brain disorder brought on by its deficiency in classic Glut1 DS. Moreover, the loss of brain neurons in mutant mice serves as a potential explanation for the deceleration of head growth and acquired microcephaly commonly observed in the human condition.

### Induction of Glut1 haploinsufficiency at later postnatal stages is associated with progressively milder Glut1 DS phenotypes.

**Figure 7:**
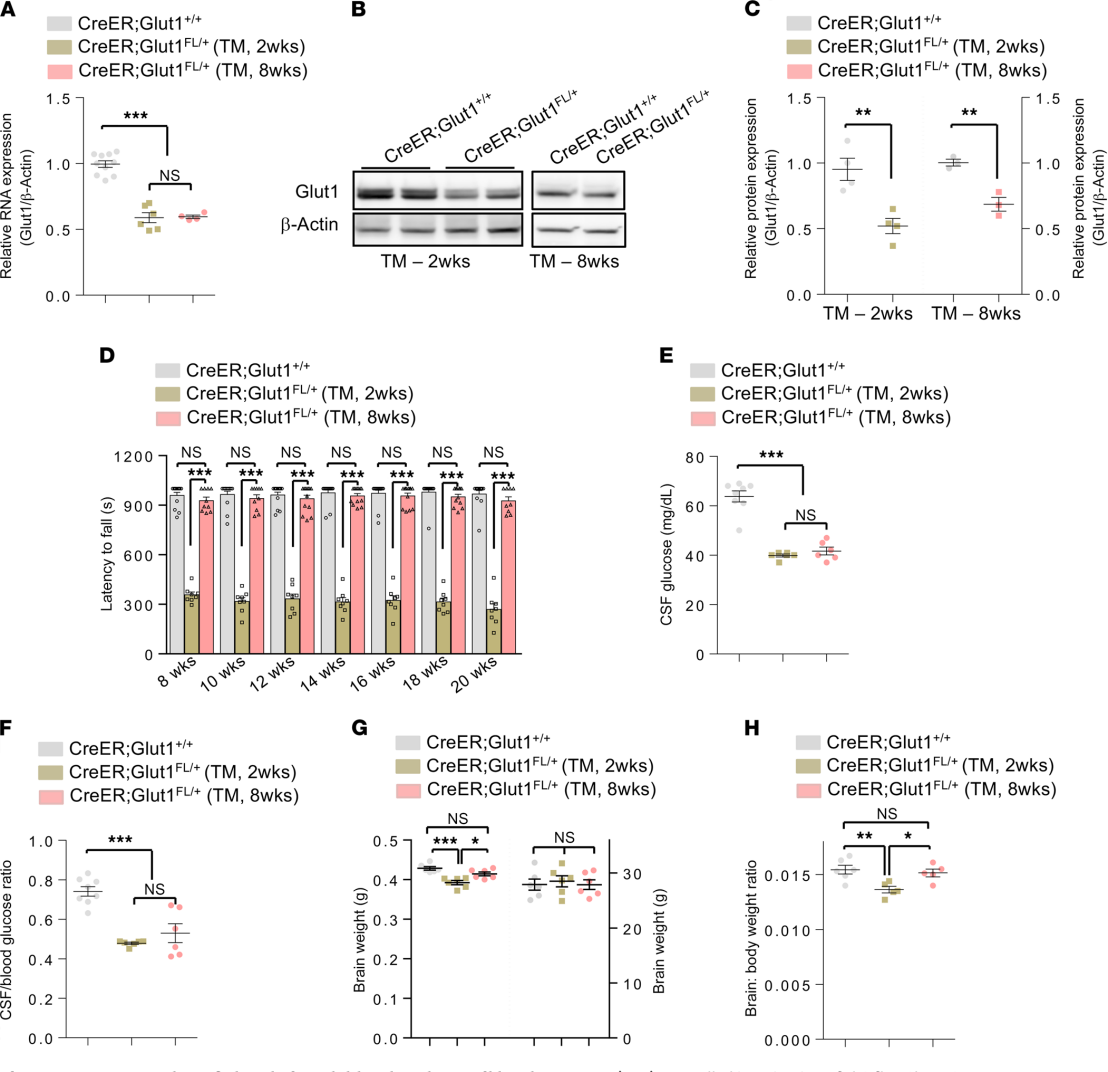
Heterozygous loss of Glut1 during adulthood produces milder phenotypes. (**A**–**C**) Controlled inactivation of the floxed *Glut1* allele at either 2 weeks or 8 weeks results in significantly lower mRNA (**A**) and *Glut1* protein (**B** and **C**) in brain tissue of 5-month-old mutant mice. ***P < 0.001, 1-way ANOVA and *t* test for **A** (*n* = 11 controls, 6 TM-2 weeks [wks], 4 TM-8wks) and **C** (*n* = 4 controls, 4 TM-2wks, 3 TM-8wks), respectively. **P < 0.01. (**D**) Inducing Glut1 haploinsufficiency in juvenile but not adult mice causes impaired performance on the rotarod, suggesting that the mature brain is protected from the adverse effects of transporter loss; ***P < 0.001, 1-way ANOVA, *n* ≥ 8 mice analyzed in each cohort. (**E** and **F**) Hypoglycorrhachia is nevertheless detected in both cohorts (**E**), as is an abnormally low CSF/blood glucose ratio (**F**); ***P < 0.001, 1-way ANOVA (*n* = 8 controls, 5 TM-2wks, 6 TM-8wks for **E** and **F**). (**G** and **H**) Micrencephaly is detected if Glut1 paucity is induced in juvenile but not adult mice. Note that body weights remain unchanged in either cohort of mutant; *P < 0.05, **P < 0.01, ***P < 0.001, 1-way ANOVA, *n* = 6 mice of each cohort analyzed.

Early postnatal Glut1 repletion with AAV vectors is a promising therapeutic strategy for Glut1 DS (12). However, the durability of such a treatment considering the episomal nature of AAV and the inevitable turnover of transduced cells, especially ECs (28), is unclear. We therefore investigated the health effects of potentially reverting to a Glut1 haploinsufficient state in spite of early postnatal repletion of protein to WT levels. This was modeled by inducing haploinsufficiency in *CreER;Glut1^fl/+^* mice at 2 additional time points — either at 2 weeks, a juvenile stage of life, or in mature, 8-week-old mice. The mutants were then assessed as previously described. Akin to depleting Glut1 at P2, we found that treating *CreER;Glut1^fl/+^* mice with TM at 2 weeks and at 8 weeks significantly reduced Glut1 transcript and protein (Figure 7, A–C). However, while the knockdown at 2 weeks significantly impaired motor performance on the rotarod, depleting Glut1 at 8 weeks did not adversely affect mice in this assay (Figure 7D). This immediately suggested a greater requirement for Glut1 in the younger mice. Inactivating both *Glut1* alleles in adult mice is reportedly fatal (29). We investigated this finding by administering TM at 8 weeks to mutants homozygous for the floxed allele (*CreER;Glut1^fl/^^fl^*). Expectedly, Glut1 transcript and protein were depleted to an even greater extent in these mice than they were in *CreER;Glut1^fl/+^* mutants (Supplemental Figure 9, A–C), and this level of depletion did result in poorer performance on the rotarod (Supplemental Figure 9D). Nevertheless, in contrast to effecting knockdown at P2 in these mutants (Supplemental Figure 5D), fatalities were minimal during the period of study and involved just 3 of 13 mice (Supplemental Figure 9E).

In spite of the overt phenotypes observed in the *CreER;Glut1^fl/fl^* mutants and since human Glut1 DS never involves complete ablation of Glut1, we limited our subsequent analyses to determining the effects of protein knockdown in the *CreER;Glut1^fl/+^* mutants. Considering the absence of any major impairment on the rotarod in mutants induced to become haploinsufficient as adults, we began by assessing CSF glucose in the 2 cohorts of mice. Consistent with significant Glut1 reduction — as assessed by transcript and Western blot analysis — we found evidence of hypoglycorrhachia in both 8-week treated and 2-week treated animals (Figure 7, E and F). However, micrencephaly was only evident in mutants depleted of Glut1 as juveniles (Figure 7, G and H) while EEG abnormalities were undetectable in either cohort (SWDs; Glut1 depleted at 2 weeks [controls = 4.5 ±4.5; mutants = 3.0 ± 1.73; *P* = 0.76, Mann-Whitney *U* test, *n* = 4]; Glut1 depleted at 8 weeks [controls = 0; mutants = 10.5 ± 10.5; *P* = 0.43, Mann-Whitney *U* test, *n* = 4]).

**Figure 8:**
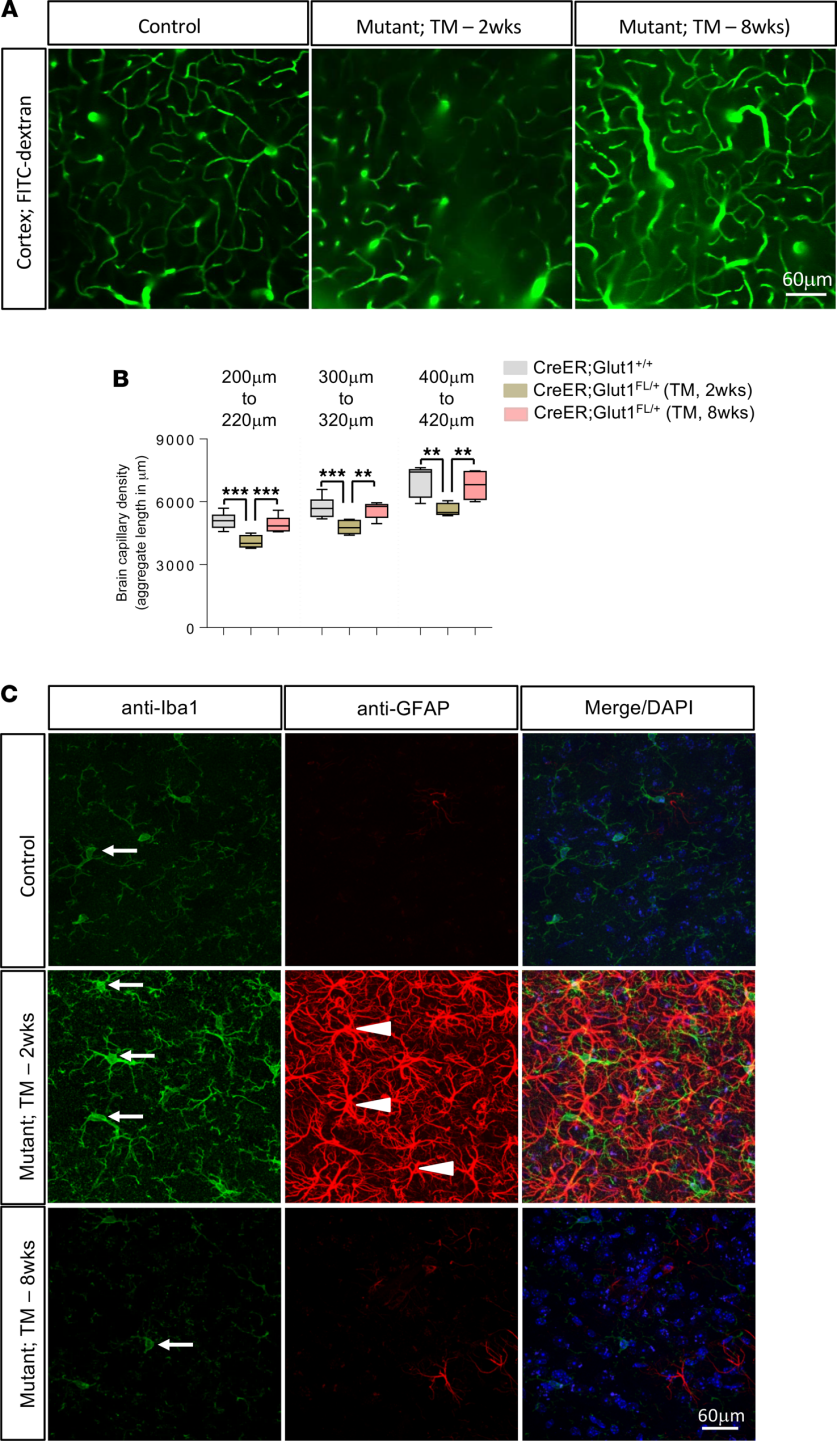
Brain microvasculature unimpaired in mutants depleted of Glut1 during adulthood. (**A**) Representative live imaging experimental result of the cortical brain microvasculature, at a depth of 400–420 μm, of 5-month-old controls and *CreER;Glut1^fl/+^* mutants wherein haploinsufficiency was induced at the indicated time points. Note reduced capillary density in mutants treated with tamoxifen at 2 weeks relative to that in controls and mutants treated at 8 weeks of age. (**B**) Graphical representation of cortical capillary densities at indicated depths in the 3 groups of mice following 2-photon live imaging; **P < 0.01, ***P < 0.001, 1-way ANOVA, *n* = 6 mice of each cohort analyzed. (**C**) Representative thalamic brain sections from 5-month-old mice belonging to the 3 cohorts stained with antibodies against Iba1 and GFAP. Neuroinflammation was only detected in *CreER;Glut1^fl/+^* mutants induced to become *Glut1* haploinsufficient as juveniles. Arrows and arrowheads depict activated microglia and astrocytes, respectively.

In a final set of analyses, we queried whether depleting Glut1 in juveniles or adults affected the brain microvasculature. Two-photon imaging of cortical brain of 5-month-old mutants showed that inducing haploinsufficiency at 2 weeks did significantly reduce overall capillary density (Figure 8, A and B). In contrast, depleting Glut1 during adulthood failed to diminish the size of the capillary network. This suggests that the mature brain microvasculature is relatively refractory to Glut1 haploinsufficiency. Consistent with these observations, we found that whereas depleting Glut1 at 2 weeks did trigger a neuroinflammatory response in 5-month-old mice, doing so at 8 weeks had no effect (Figure 8C). The gliosis in the latter cohort only became evident at 12 months of age (Supplemental Figure 10), suggesting that despite the formation of a normal capillary network, persistent hypoglycorrhachia does eventually activate glia. Still, in combination with the outcome of the experiments on mutants depleted of Glut1 during neonatal life, the collective data on the CreER mice suggest that the organism becomes increasingly resistant to Glut1 haploinsufficiency as it transitions from neonatal life through juvenile stages and into adulthood.

## Discussion

Although relatively rare, Glut1 DS serves as a useful paradigm for the study of myriad human conditions. In part, this stems from the numerous biological pathways impacted directly or indirectly by the Glut1 protein. Indeed, Glut1 is involved in disorders as diverse as Alzheimer’s disease, retinitis pigmentosa, cancer and diabetes (7). Here, we extend our understanding of the consequences of perturbing Glut1 through the study of the disease most directly associated with the transporter — Glut1 DS. Five main findings of biological and clinical import emerge from our study. Chief among these is the discovery that Glut1 functions cell autonomously in ECs to cause Glut1 DS. Selectively depleting the transporter in ECs not only resulted in, more or less, the entire gamut of cellular and behavioral phenotypes associated with the human condition, but it also revealed potentially novel defects in a select population of these cells — tip cells, which are crucial for brain angiogenesis. A second notable result reported here is the profound neuroinflammation within the Glut1 DS brain. Considering how rapidly it emerged — within the first week of life — the gliosis, triggered even when glia remained WT for Glut1, is likely a primary driver of disease pathology. A third important discovery is that low Glut1, even if restricted to ECs, results in correspondingly low levels of BDNF. Given what is known about BDNF function, the neurotrophin is a potentially important mediator of Glut1 paucity and one reason for selective brain dysfunction in Glut1 DS. A fourth salient finding is that of fewer brain neurons in Glut1 DS — an intuitively appealing explanation for the characteristic micrencephaly observed in the disease. The reduction in neuron numbers is at least in part a result of a non–cell-autonomous effect of Glut1 haploinsufficiency in brain endothelia. Finally, our study casts important light on the temporal requirements for the Glut1 protein. Scarcity of the transporter was most deleterious when haploinsufficiency was induced early in life. Vulnerability to low protein declined with age, suggesting that Glut1 is particularly important for processes active during infancy or childhood.

We previously reported on brain angiogenesis defects in model mice constitutively haploinsufficient for *Glut1* (12). However, since Glut1 is expressed not just in the brain microvasculature, but also in astrocytes, the cellular origin of the abnormality and indeed the wider Glut1 DS phenotype remained unclear. The current study addresses this. We show that ECs constitute a critical cellular site of action of the Glut1 protein by demonstrating that selective loss of the transporter in these cells is sufficient to not only trigger brain angiogenesis defects, but also to cause overt disease. Indeed, *Tie2-Cre;Glut1^fl/+^* mice exhibited all of the signature features of Glut1 DS observed in mutants constitutively haploinsufficient for Glut1. The identification of abnormalities in endothelial tip cells furthermore establishes the cellular basis of the diminished brain microvasculature in Glut1 DS. These cells are critically important for angiogenesis (30) but exist in an avascular microenvironment containing limited (approximately 1 mM) supplies of extracellular glucose as an energy source (31). Crucially, they are almost totally reliant on glycolysis to fuel their proliferation (32). A relatively modest (~35%) decrease in glycolytic flux within them prevents the formation of capillary sprouts, diminishes proliferation, and promotes quiescence. If, as suggested by Barros et al. (33), Glut1 haploinsufficiency results in a 90% drop in extracellular brain glucose, the reduction from 1 mM to 0.1 mM of the nutrient would devastate tip cells. Our findings of reduced numbers of tip cells with stunted lamellipodia in constitutively haploinsufficient mutants are entirely consistent with this scenario. Considering the period of postnatal development — the first 2 weeks of life — when tip cells actively proliferate (14), it is also not surprising that controlled depletion of Glut1 at P2 affected them and, thus, overall brain angiogenesis. Expectedly, inducing haploinsufficiency at 2 weeks or 8 weeks — once tip cells have stopped proliferating — produced mild or minimal diminution of the brain microvasculature.

Our results also suggest that glial activation constitutes an important aspect of overall Glut1 DS brain pathology. Such pathology has received little attention in the literature but might have been expected, given the relatively high expression of Glut1 in astrocytes. However, to our surprise, neuroinflammation was not only triggered non–cell autonomously by low Glut1 in ECs, but it was also among the earliest discernible cellular defects in the mutant. The fact that the gliosis appeared at 1 week of age when we were unable to detect any difference in the cerebral microvasculature of mutant and control mice (12) suggests that brain neurons in the mutant become distressed almost immediately after birth. Still, the nature of the neuroinflammatory response — adaptive or maladaptive — is currently unclear, as is the sequence in which glia become activated. The role of microglia in activating proinflammatory A1 astrocytes and initiating the overall response has recently been described (34), but whether this also is the case in Glut1 DS — or if the initial response is adaptive involving antiinflammatory glia — remains to be determined. At any rate, the gliosis becomes more pronounced with time and likely eventually turns maladaptive, retarding synapse formation and perhaps even prompting cell loss. The paucity of thalamic neurons we observed in adult mutant mice is consistent with this hypothesis. Future studies will explore this idea. Still, the combination of neuroinflammation, cell loss, and reduced BDNF levels that we discovered in this study begins to inform us as to how depletion of the widely expressed Glut1 protein causes selective brain dysfunction. Among these various findings, the effect of Glut1 scarcity on BDNF levels is especially noteworthy. BDNF is an established mediator of proper neuron function, modulating synaptic plasticity, neuronal differentiation, and survival and, consequently, affecting behavioral parameters such as memory and learning (25, 26). Intriguingly, it is also reported to mediate angiogenesis (35). Low levels of this factor could therefore have dual implications in Glut1 DS — retarding brain angiogenesis on the one hand and acting directly on neurons to compromise their health and viability on the other. Precisely how low Glut1 affects BDNF depletion in Glut1 DS is presently unclear but likely involves reduced glycolytic flux and, thus, reduced lactate. Lactate was recently reported to induce BDNF expression via the transcriptional coactivator PGC1α and the myokine FNDC5 (27). Paucity of this key glycolytic metabolite could therefore result in correspondingly low BDNF.

Additional factors purportedly underlying brain microvasculature diminution in Glut1 DS have recently been reported (17). These include AMPK and its various downstream effectors, among them the cell-cycle inhibitor p53. However, the study by Veys and colleagues that implicated these factors in Glut1 deficiency failed to consider a critically important aspect of the human disorder — that it is a consequence of *SLC2A1* haploinsufficiency — and instead drew its inferences about disease mechanisms from cultured cells and mice engineered to be completely devoid of Glut1. While conclusions arrived at in this manner regarding Glut1-associated mechanisms may nevertheless be instructive from a basic biology standpoint, we think the mechanisms — and indeed most observations made under conditions of total Glut1 ablation, which is embryonically lethal (19) — can mislead if extrapolated to suggest relevance to the haploinsufficient state in Glut1 DS. Indeed, we were unable to detect any evidence of altered phospho-AMPK, suggesting that a reduction of Glut1 by half does not trigger changes in the expression of this factor. Our analysis also failed to detect evidence of an increase in activated p53. Instead, Glut1 haploinsufficiency reduced levels of a number of p53 target genes. The physiological relevance of such repression and the implied reduction in p53 activity remains to be investigated.

This study also delineates the temporal requirements for the Glut1 protein, critical to implementing future treatments for Glut1 DS and ensuring optimal therapeutic outcomes. Such information is especially pertinent, considering the results of preclinical studies that raise the prospect of gene replacement as a means of treating the human condition (12, 36). Consistent with the pediatric nature of Glut1 DS and reminiscent of the outcome of similar studies (37, 38) on another infantile-onset disease, spinal muscular atrophy, the earlier we effected Glut1 haploinsufficiency in our conditional mutants, the more closely we were able to mimic disease in constitutively haploinsufficient mutants and in human patients. These observations emphasize an early requirement for the Glut1 protein and imply a limited therapeutic window of opportunity for the human disease. From a clinical standpoint, this means that it will be important to institute newborn screening to identify the genetically affected clinically presymptomatic infant and treat as early as possible. However, our evidence of an increased resistance to low Glut1 as the organism matures also raises the possibility that treatments, particularly gene replacement, may not have to be repeatedly administered to ensure a disease-free state. Still, one concern stems from the late-onset neuroinflammation observed in mice modeled to test how the afflicted individual who has undergone early Glut1 augmentation might respond if she or he lost cells restored for the protein. Potential long-term consequences of such brain pathology particularly in humans will have to be carefully considered. In spite of these and other important questions that might be raised, the information gleaned from our study will be important as we continue to pursue effective treatments for brain energy failure syndromes such as Glut1 DS.

## Methods

### Mice.

Study subjects were maintained on a 129 S6/SvEvTac genetic background. Animals bearing the *Glut1*^Δ^ allele were generated as previously described (16). Mice harboring the Tie2-Cre (stock no. 008863), Zp3-Cre (stock no. 003651), and CreER (stock no. 008463) drivers were obtained from the Jackson Laboratory, backcrossed over 6 generations to the 129 S6/SvEvTac genetic strain background and genotyped using primers described in Supplementary Information (SI). Note that although the Tie2-Cre line is an established tool to deplete proteins in ECs, it is also reported to express Cre in a limited subset of non-ECs, notably macrophages. TM administrations first involved preparing stock solutions (20 mg/mL or 50 mg/mL) of the compound (T5648, MilliporeSigma) in 100 μL of ethanol and 900 μL corn oil. Three doses of 250 mg/kg (to 2-week-old mice) and 450 mg/kg (to 8-week-old mice) were delivered by oral gavage over consecutive days to the mice. P2 mice received 2 doses (125 mg/kg) of the TM on consecutive days. Subsequent analyses were then conducted at either 2 weeks of age (brain microvasculature studies) or at approximately 5 months of age (remaining assessments).

### Phenotypic evaluations and CSF/blood glucose measurements.

For the behavioral studies, GraphPad Prism was used to determine sample sizes to detect differences of at least 2 SDs with a power of 80% (*P* < 0.05). Mice were not randomized, but as mutants do not exhibit an overt disease phenotype, it was possible to blind the investigator to the particular cohort being assessed. Rotarod tests were performed on an accelerating rotarod (Ugo Basile Inc.) as described in SI. To assess brain and body size, and investigate CSF/serum glucose levels, mice were first subjected to an overnight fasting period. Subsequently, they were weighed, blood was collected from the tail vein, CSF was extracted from the cisterna magna as detailed by us in prior studies (12, 19), and the brain was removed and weighed. Glucose concentrations in the CSF and blood were assessed on disposable strips using a Contour Next EZ glucose meter (Bayer Corp.). Except for the determination of brain and body weights where results presented are from male mice alone, experiments described in this study included animals of either sex.

### Quantitative PCR, Western blots, and preparation of brain capillary fractions.

Tissues were lysed using TRIzol (Thermo Fisher Scientific) according to the manufacturer’s instructions. Following RNA extraction and cDNA synthesis, quantitative PCR (qPCR) was performed in triplicate on a CFX96 Real-Time PCR machine (Bio-Rad). Primer sequences are presented in SI. Glut1, phospho-AMPK, and p53 protein levels were assessed by Western blots using antibodies detailed in SI and procedures previously described (12). Protein bands were visualized on an ImageQuant LAS 4000 machine (NA-931, GE Healthcare) using the ECL Detection Kit (RPN 2109, GE Healthcare). Band intensities were determined using ImageJ software (NIH). Brain parenchymal and microvasculature fractions were separated using a modified version of a protocol described by Galea et al. (39). Briefly, fractions were separated on an 18% dextran gradient and either processed immediately or stored at –80°C until required. For details, see SI.

### AAV9 delivery of Glut1 to mice.

The murine *Slc2a1* gene was packaged into an single-stranded AAV9 vector harboring a CMV-enhanced chicken β-actin promoter and AAV2 inverted terminal repeats (ITRs) flanking the expression cassette as reported in a previous study (12). Delivery of the AAV9-Glut1 vector (4.2 × 10^11^ GC in 35 μL) into P1 mice was accomplished through the retro-orbital sinus.

### IHC and cell counts.

Mice were perfused with 1× PBS and then 4% PFA, before incubating whole brains overnight in 4% PFA. Coronal sections 50 μm thick were cut 24 hours later on a vibratome (LEICA VT1000 S), incubated (1 hour at room temperature [RT]) in blocking solution (3% BSA, 0.5% Triton X-100 in PBS) before further incubating them with primary and secondary antibodies (see SI). The sections were then washed 3 times (20 minutes each) with 1× PBS, mounted with Vectashield (Vector Laboratories) on slides, and overlaid with coverslips for microscopic analysis. Brain angiogenesis was assessed in 5-month-old mice as previously described (12). Briefly, brain sections were stained with fluorescein-conjugated *Lycopersicon* *esculentum* lectin (Vector Labs), and 20 μm stack images of the thalamus were acquired using a LEICA TCS SP8 confocal microscope. Capillary density involved quantifying the aggregate length of vessels < 6 μm in diameter in a 184 μm × 184 μm area. Three such areas, nonadjacent to one another, in each of at least 3 animals were examined. Images presented in the manuscript are reconstructions of 3 dimensional *Z*-stacks. It is important to note that mean capillary length in relevant figures refers to the average length of the lectin or Glut1-stained blood vessels; total or aggregate length denotes the sum total distance of the stained vessels in the *Z*-stacks. Tip cells were assessed and quantified in cortex and thalami of P14 mice. Sections prepared as described above were stained for 48 hours (4°C) with a primary antibody that recognized Glut1; they were washed and then incubated with an Alexa 488–conjugated goat anti-mouse secondary antibody for a further 12 hours (4°C). Sections were then washed again and mounted in Vectashield (Vector Labs Inc.), and 30 μm stack images were acquired at a magnification of 63× on a LEICA TCS SP8 confocal microscope. Tip cells were analyzed using the LAS AF Lite software suite (Leica Inc.) in ≥ 3 slices per animal; 3 fields per slice were examined. Inclusion criteria for tip cells specified > 3 lamellipodia, each of which had to be at least 5 μm in length. Gliosis in the mice was examined and quantified in 15 μm stack images of the ventral posteromedial (VPM) thalamic nuclei using antibodies against GFAP and Iba1. Sections were prepared and stained, and images were acquired as described above. Quantification was carried out on 63× images. Neurons in the VPM thalamic nuclei were visualized with an antibody against NeuN and similarly quantified with the exception that analyses were conducted on 10 μm *z*-stack images. BDNF^+^ cells were also assessed in the VPM thalamic nuclei as described for NeuN^+^ cells. All quantification was carried out by investigators blinded to mouse genotype. Additional details on antibodies and dilutions employed are supplied in the SI section.

### In vivo, 2-photon imaging of brain capillaries.

To image the cerebral microvasculature in live animals, we followed a protocol originally described by McCaslin et al. (40), subsequently refined (41, 42) and then employed by us in a previous study (12). For details, see SI.

### EEG analysis.

Mice aged 4–5 months were anesthetized (250 mg/kg 2,2,2-Tribromoethanol [MilliporeSigma, T48402]), burr holes were drilled through the skull only, and silver wire electrodes were placed over the left and right frontal cortices and left posterior cortices. A reference electrode was placed over the cerebellum. The wires were soldered to a microconnector, and the assembly was secured to the skull with zinc oxide cement. EEG recordings were carried out after a 48-hour (or longer) recovery period. Mice were connected either to a Natus Quantum 128 (Natus Medical Inc.) or Grael 4k (Compumedics Ltd.) programmable amplifier, and activity was video recorded for either 24 or 48 hours simultaneously using a night vision–enabled SONY EP550 camera. Differential amplification recordings were recorded pairwise between each recording electrode and the reference. EEG data were visualized and analyzed for SWD with interactive seizure detection software (Assyst 2.0; Kaoskey Inc.) used to facilitate detection, for which the right frontal electrode was used (i.e., against reference) — although when SWDs were called, they were typically observed at similar amplitude in the left frontal channel and either absent from or present at much lower amplitude in the left posterior channel. To be called a SWD, an event must consist of repeated wave-spike complexes (of at least 3 beats) lasting 0.5 seconds or longer, in the typical range for rodents (5.5–9 Hz) and at least twice the amplitude of nearby baseline EEG.

### Statistics.

The unpaired 2-tailed Student’s *t* test with Welch’s correction, Mann-Whitney *U* test, or 1-way ANOVA followed by Tukey’s post hoc comparison, where indicated, were used to compare means for statistical differences. Kaplan-Meier survival curves were assessed for differences using the long-rank test equivalent to the Cochran-Mantel-Haenszel test. Data in the manuscript are represented as mean ± SEM unless otherwise indicated. *P* < 0.05 was considered significant. Statistical analyses were performed with GraphPad Prism v6.0.

### Study approval.

All animal procedures adhered to protocols described in the *Guide for the Care and Use of Laboratory Animals* (National Academies Press, 2011) and were approved by Columbia University’s IACUC. The subjects of this study were randomly selected 129 S6/SvEvTac male and female mice housed in a controlled environment on a 12-hour light/dark cycle with food and water.

## Author contributions

MT planned and performed most of the experiments described here. SHP and FL carried out the immunostaining and mouse behavior studies, respectively. SP assisted with the EEG analysis, and WNF supervised this aspect of the project. HY and CYK performed the in vivo imaging studies, while EMCH supervised these experiments. CBR, SJ, EDA, LD, and YL provided intellectual input and reagents. DCDV provided intellectual input and helped prepare the manuscript. URM conceptualized the experiments, directed the project, analyzed and interpreted the data, and wrote the manuscript.

## Supplementary Material

Supplemental data

## Version 1 12/22/2020

In-Press Preview

## Version 2 02/08/2021

Electronic publication
