# Supplementary material for: An early endothelial cell–specific requirement for Glut1 is revealed in Glut1 deficiency syndrome model mice
Source: JCI Insight. 2021 Feb 8;6(3):e145789. doi: 10.1172/jci.insight.145789 (PMC7934852; doi:10.1172/jci.insight.145789)
Supplement: Supplemental data [file jciinsight-6-145789-s221.pdf]

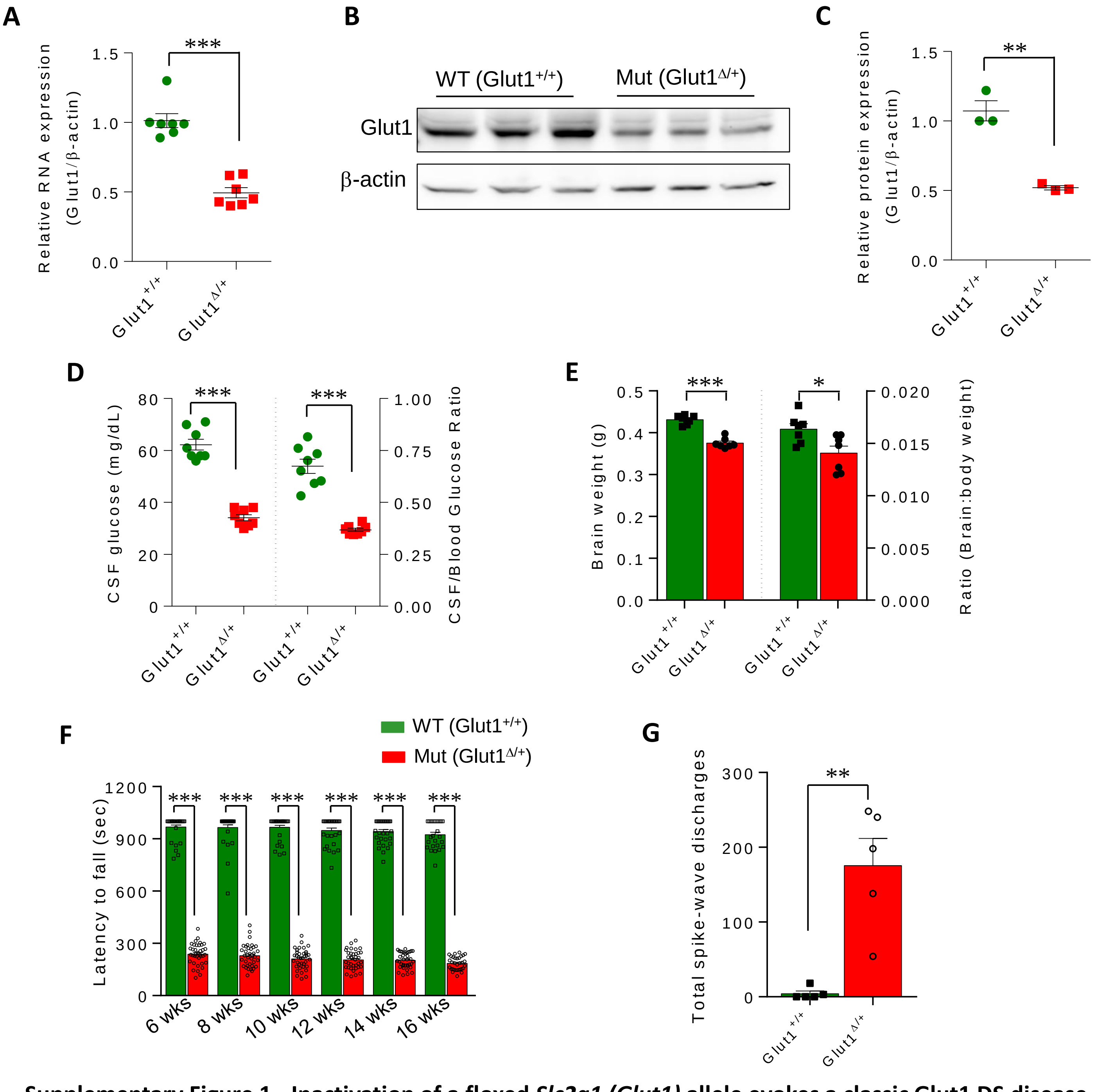

Supplementary Figure 1 - Inactivation of a floxed *Slc2a1 (Glut1)* allele evokes a classic Glut1 DS disease phenotype. (A) Graph depicts reduced Glut1 transcript in brain tissue of *Glut1*$^{Δ/+}$ mutants. ***, $P < 0.001$, *t* test, *n*=7 mice of each cohort. **(B)** Western blot and **(C)** quantified result of blot depicting reduced Glut1 protein in brain of *Glut1*$^{Δ/+}$ mutants. **, $P < 0.01$, *t* test, *n*=3 mice of each cohort. Evidence of **(D)** hypoglycorrhachia, **(E)** micrencephaly and **(F)** poor motor performance on the rotarod in *Glut1*$^{Δ/+}$ mutants. **(G)** EEG abnormalities in the form of spike-wave discharges become evident in *Glut1*$^{Δ/+}$ mutants. Note: *, **, ***, $P < 0.05$, $P < 0.01$, $P < 0.001$ respectively, *t* test; *n*=8 (panel D), *n*=7 (panel E), *n*≥30 (panel F) and *n*=5 (panel G).

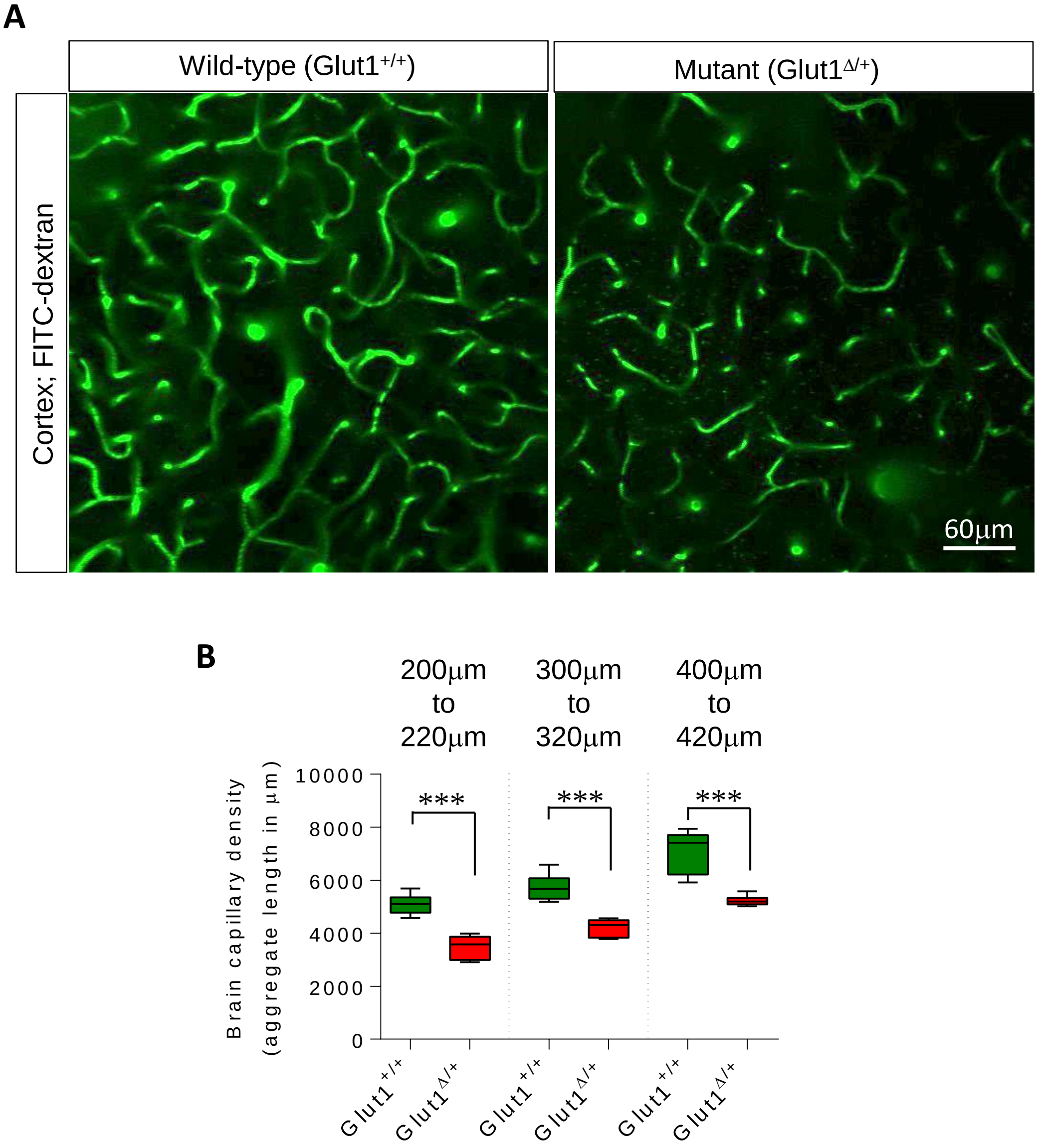

Supplementary Figure 2 – Fewer brain capillaries in *Glut1*$^{\Delta/+}$ mutant mice. (A) Representative live-imaging experimental result of the cortical brain microvasculature, at a depth of 200μm to 220μm, of a mutant and a control mouse. Note reduced density of capillaries in the mutant. **(B)** Graphical representation of cortical capillary densities at indicated depths in the two groups of mice following 2-photon live imaging; ***, $P < 0.001$, *t* test, $n \geq 9$ regions from each of N=6 mice of each cohort.

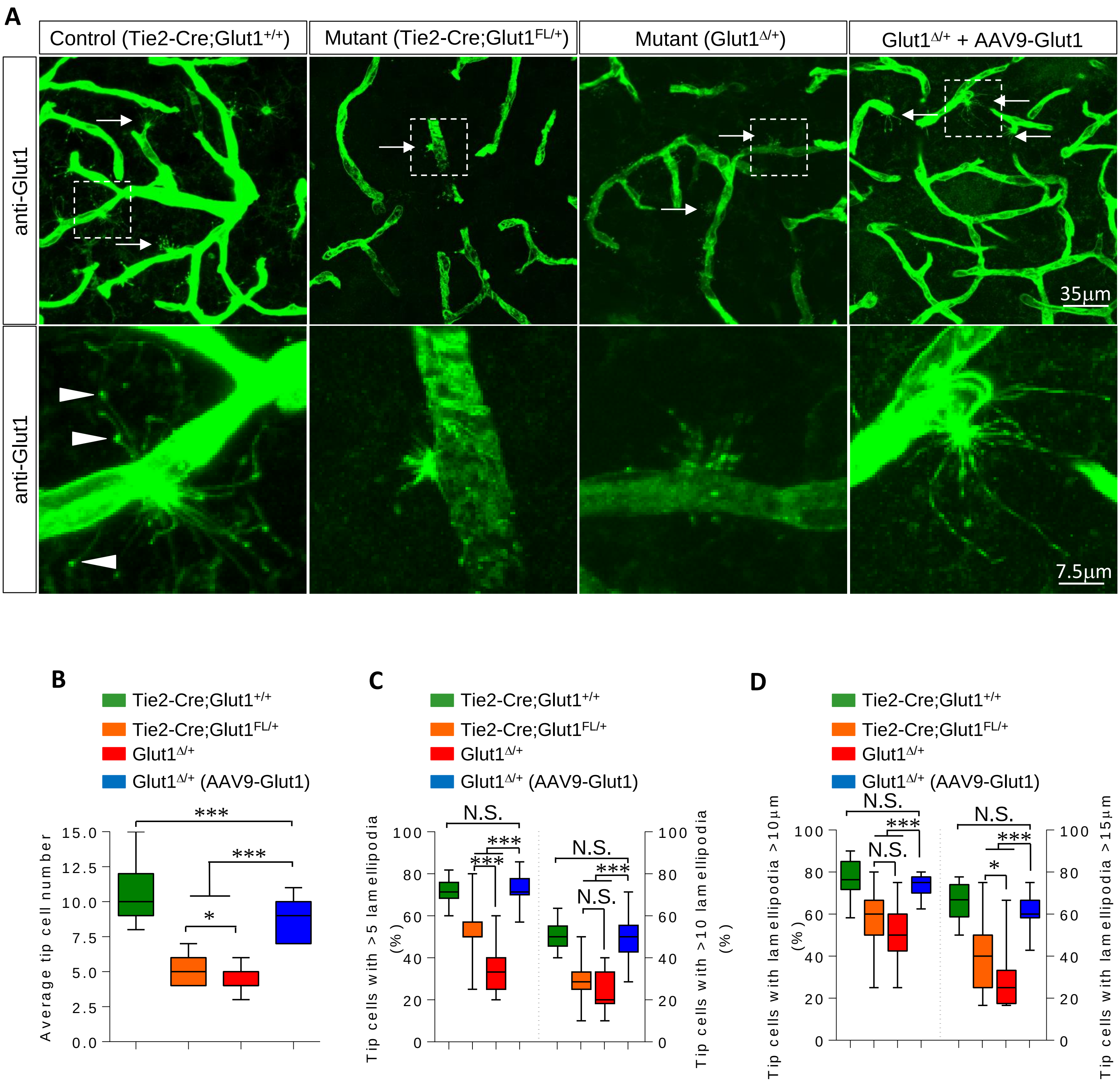

Supplementary Figure 3 – Endothelial tip cell pathology in constitutive and EC-specific Glut1 DS mutants. (A) Tip cell defects are evident in Glut1-stained cortical sections of PND14 *Glut1*$^{\Delta/+}$ and *Tie2-Cre;Glut1*$^{FL/+}$ mutants but rescued following repletion of the protein; tip cells indicated by arrows. Also depicted are magnified images of representative tip cells from each of the mice. Note the especially intense Glut1 staining at termini of control tip cell (arrowheads). Tip cell numbers are **(B)** reduced and have **(C)** significantly fewer and **(D)** shorter lamellipodia in *Glut1*$^{\Delta/+}$ and *Tie2-Cre;Glut1*$^{FL/+}$ mutants than they do in healthy controls or mutants restored for Glut1. Note: *,***, $P < 0.05$ and $P < 0.001$ respectively, *one-way ANOVA*, $n \geq 9$ regions from each of N=3 mice of each genotype examined for panels B – D.

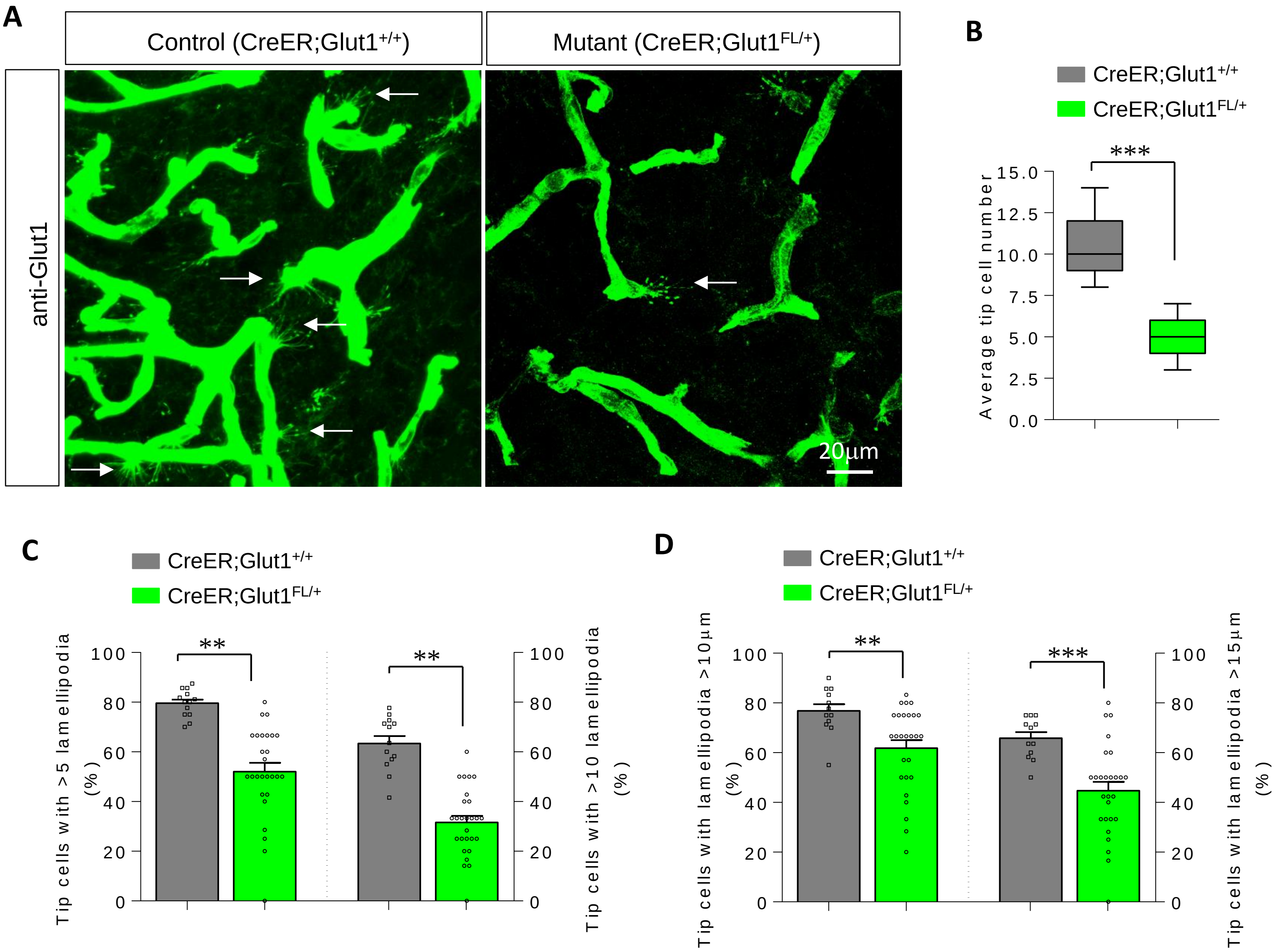

Supplementary Figure 4 – Endothelial tip cell defects in thalamic brain of mice depleted of Glut1 at PND2. (A) Thalamic brain sections of PND14 mice stained for Glut1 reveal a relative paucity and defects of mutant endothelial tip cells (arrows). Quantified results of **(B)** tip cell numbers, **(E)** lamellipodia counts and **(F)** lamellipodia size in mutants and controls. Note: **, ***, $P < 0.01$, $P < 0.001$ respectively, *t* test, $n \geq 9$ regions from each of N=3 mice of each genotype examined for panels B – D.

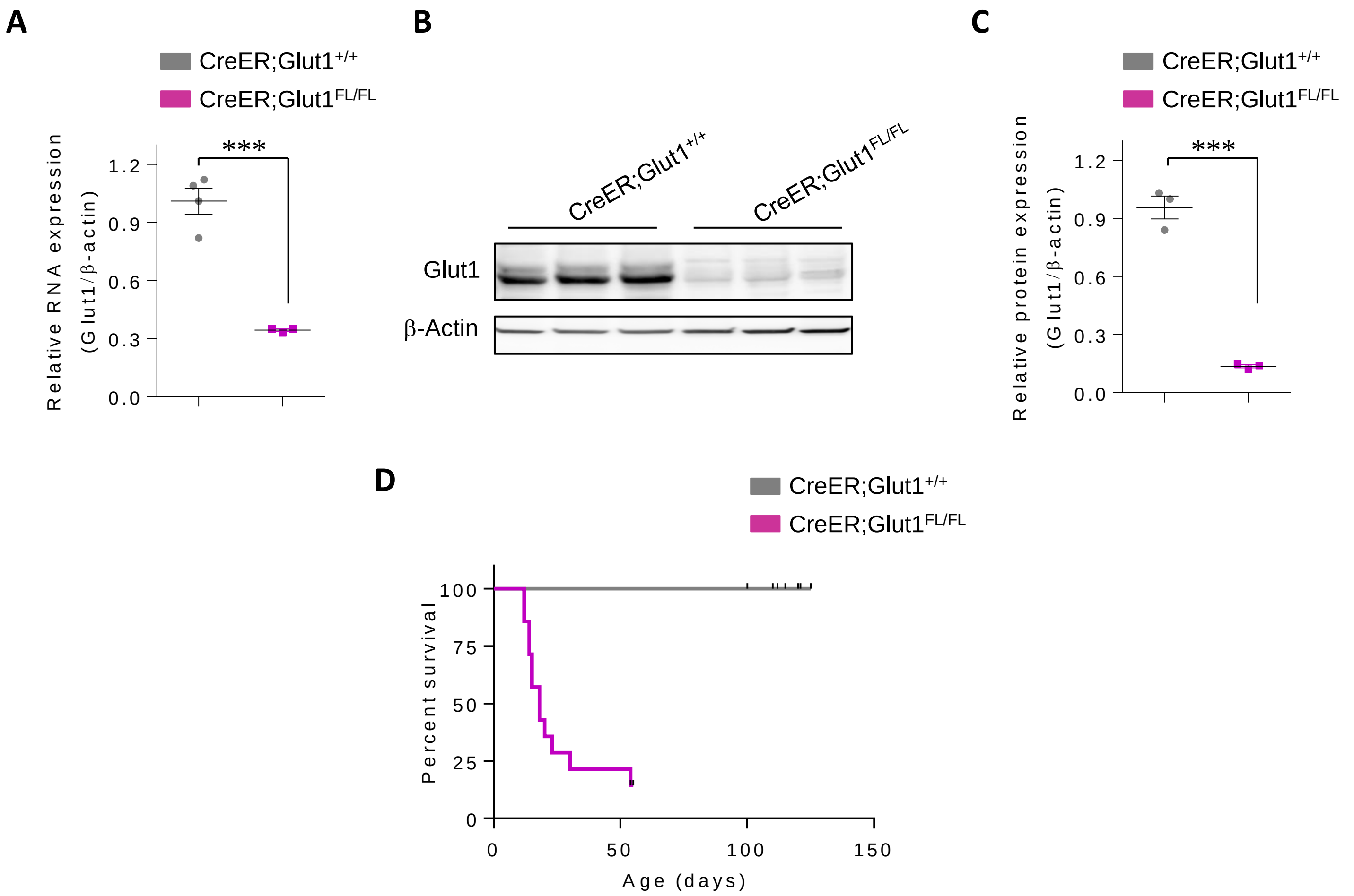

Supplementary Figure 5 – Early postnatal Glut1 depletion below heterozygous levels affects the viability of model mice. (A) Graph depicts reduced Glut1 transcript in brain tissue of treated *CreER;Glut1$^{FL/FL}$* mutants; ***, $P < 0.001$, *t* test, *n*=4 controls and 3 mutant mice analyzed. **(B)** Western blot and **(C)** quantified result of blot depicting reduced Glut1 protein in brain tissue of *CreER;Glut1$^{FL/FL}$* mutants; ***, $P < 0.001$, *t* test, *n*=3 mice analyzed. **(D)** Kaplan-Meier survival curves depict a marked reduction in lifespan of *CreER;Glut1$^{FL/FL}$* mutants depleted of Glut1 below heterozygous levels at PND2; $P < 0.0001$ between groups, Log-rank test, *n*=10 mice from each group.

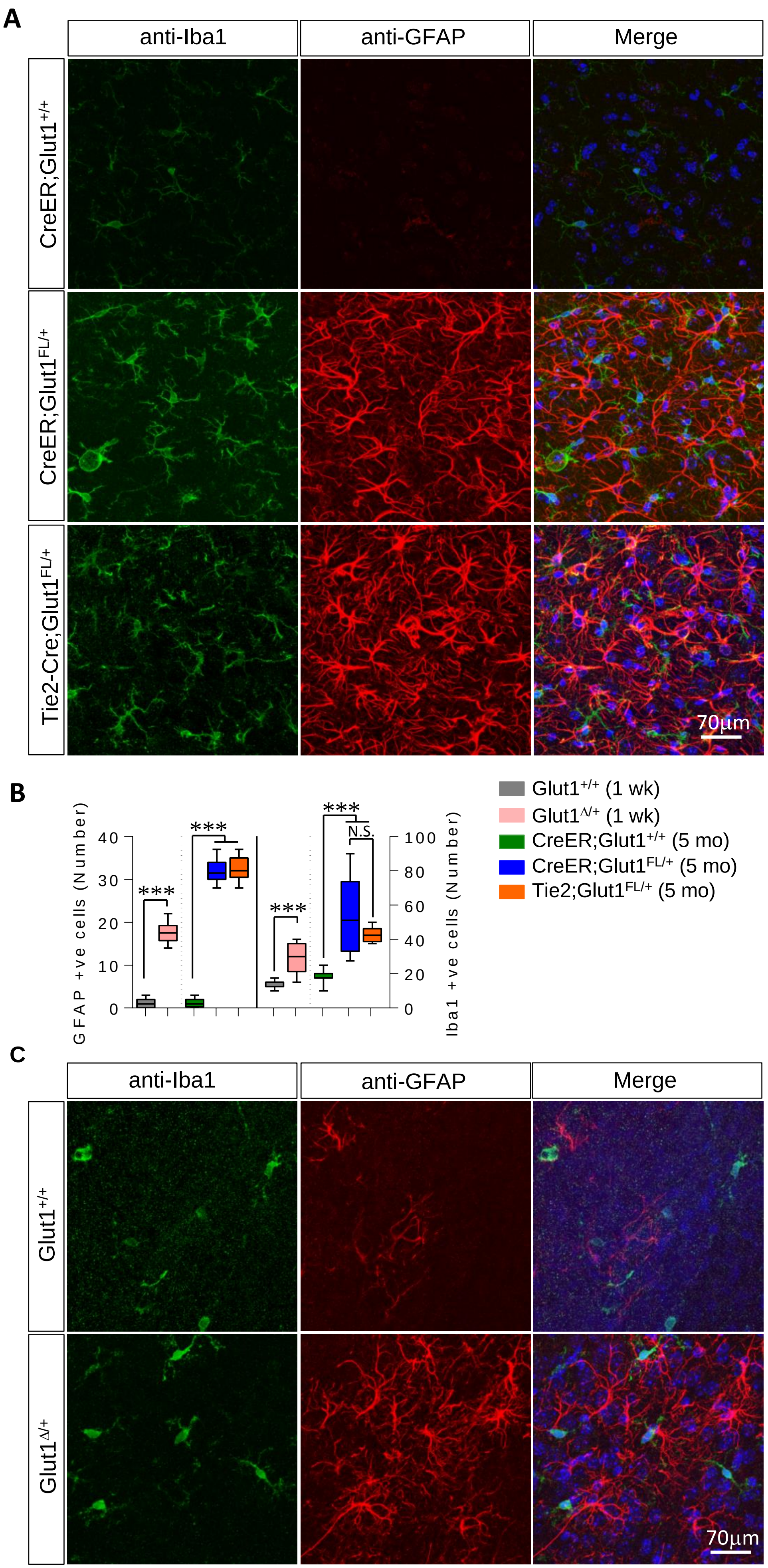
A
anti-Iba1
anti-GFAP
Merge
CreER;Glut1+/+
CreER;Glut1FL/+
Tie2-Cre;Glut1FL/+
70µm
B
GFAP +ve cells (Number)
Iba1 +ve cells (Number)
0
10
20
30
40
0
20
40
60
80
100
***
***
***
***
N.S.
Glut1+/+ (1 wk)
Glut1Δ/+ (1 wk)
CreER;Glut1+/+ (5 mo)
CreER;Glut1FL/+ (5 mo)
Tie2;Glut1FL/+ (5 mo)
C
anti-Iba1
anti-GFAP
Merge
Glut1+/+
Glut1Δ/+
70µm

Supplementary Figure 6 – Glut1 haploinsufficiency triggers early-onset brain neuro-inflammation in model mice. (A) Severe gliosis featuring activated microglia and reactive astrocytes in thalamic brain tissue (ventral posteromedial nucleus – VPM) of 5-month old mutants ubiquitously depleted of Glut1 at PND2 or selectively depleted of the protein in ECs during embryonic development. **(B)** Enumeration of reactive astrocytes and activated microglia; ***, $P < 0.001$, *t* test or one-way ANOVA, $n \geq 9$ thalamic regions from each of $N=3$ mice of each genotype assessed. **(C)** Neuro-inflammation was detected as early as 1 week of age in $Glut1^{\Delta/+}$ mutants.

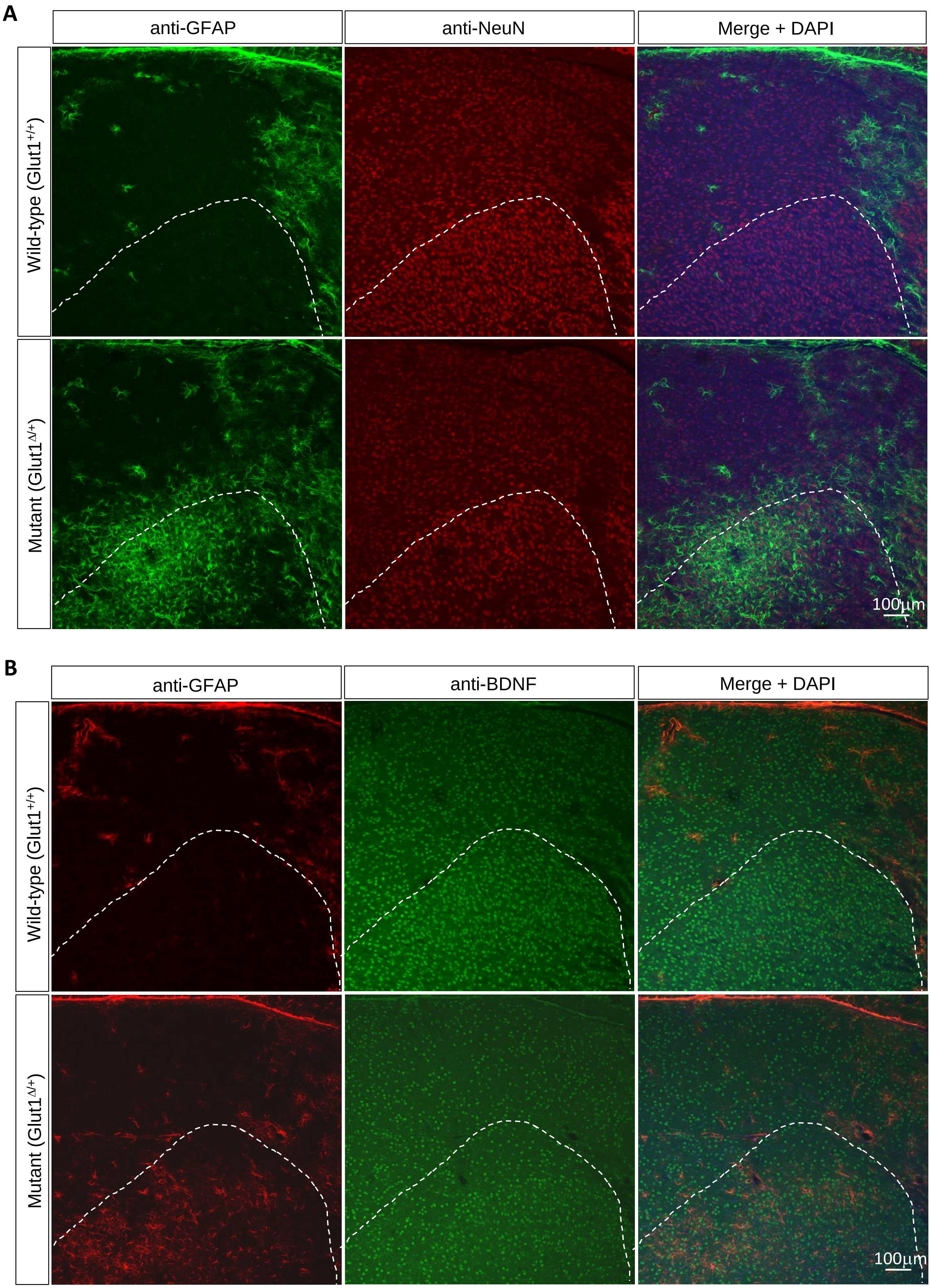

A
anti-GFAP
anti-NeuN
Merge + DAPI
Wild-type (Glut1$^{+/+}$)
Mutant (Glut1$^{\Delta/+}$)
100μm
B
anti-GFAP
anti-BDNF
Merge + DAPI
Wild-type (Glut1$^{+/+}$)
Mutant (Glut1$^{\Delta/+}$)
100μm

Supplementary Figure 7 – Glut1 DS is characterized by a deficiency of thalamic neurons. (A) Representative immuno-histochemically stained brain sections illustrate the correlation between the especially prominent astrocytosis of the mutant ventral posteromedial (VPM) thalamic nucleus (demarcated area) and the reduced numbers of neurons within this nucleus. **(B)** Sections from the same region of the brain sampled in panel (A) reveal fewer BDNF-positive cells in the mutant mouse.

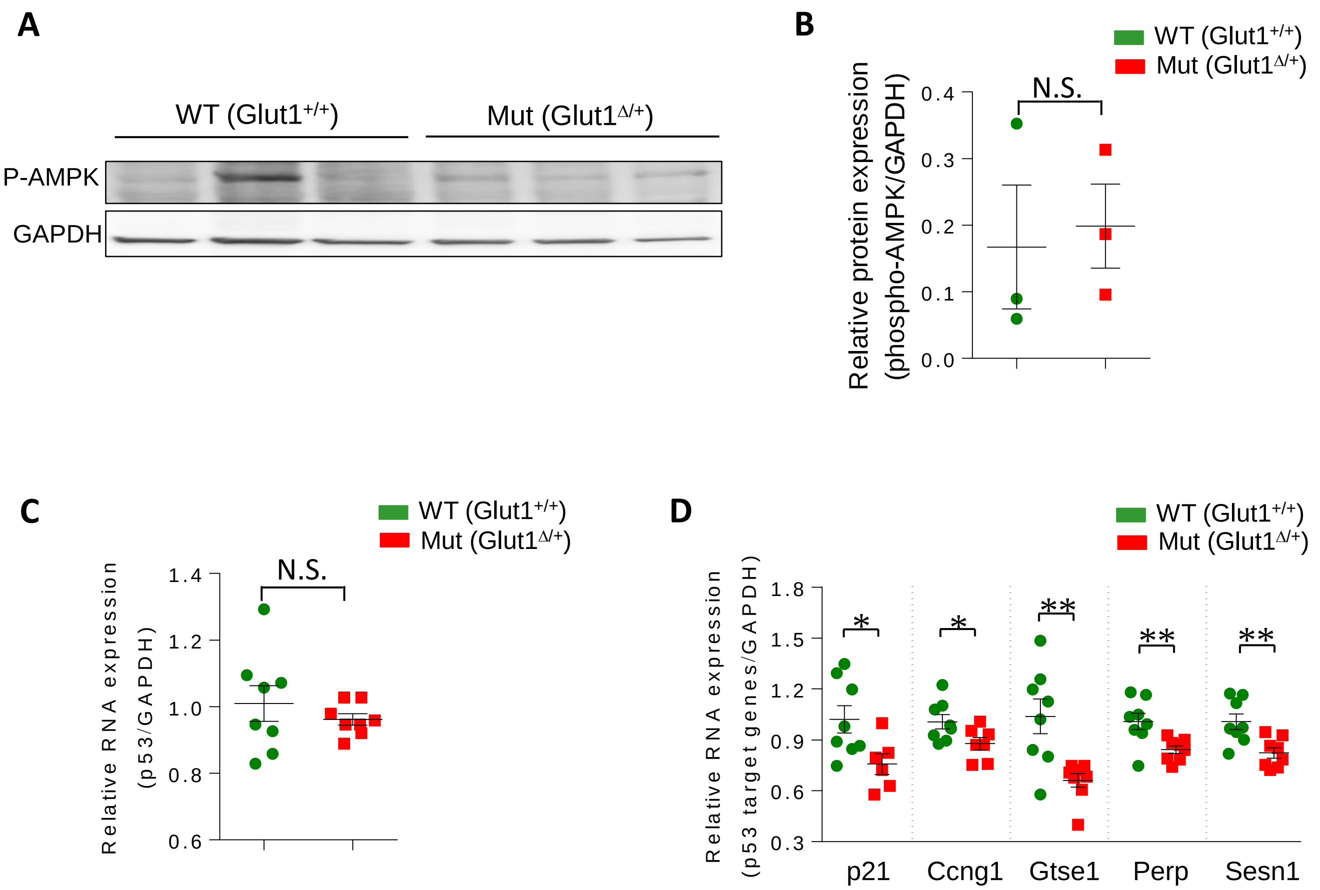

Supplementary Figure 8 – Glut1 haploinsufficiency does not alter AMPK levels or induce p53. (A) Western blot of phospho-AMPK levels in brain tissue from adult *Glut1*$^{\Delta/+}$ mutants and *Glut1*$^{+/+}$ controls. **(B)** Quantified results of western blot in panel A. Note: N.S. denotes $P > 0.05$, *t* tests, n=3 mice of each cohort. **(C)** p53 transcript levels remain unchanged in brain tissue of *Glut1*$^{\Delta/+}$ mutants. **(D)** RNA levels of p53 transcriptional targets are reduced in brain tissue of *Glut1*$^{\Delta/+}$ mutants. *, **, $P < 0.05$, $P < 0.01$, *t* tests, *n*=8 mice of each genotype assessed in panels C, D.

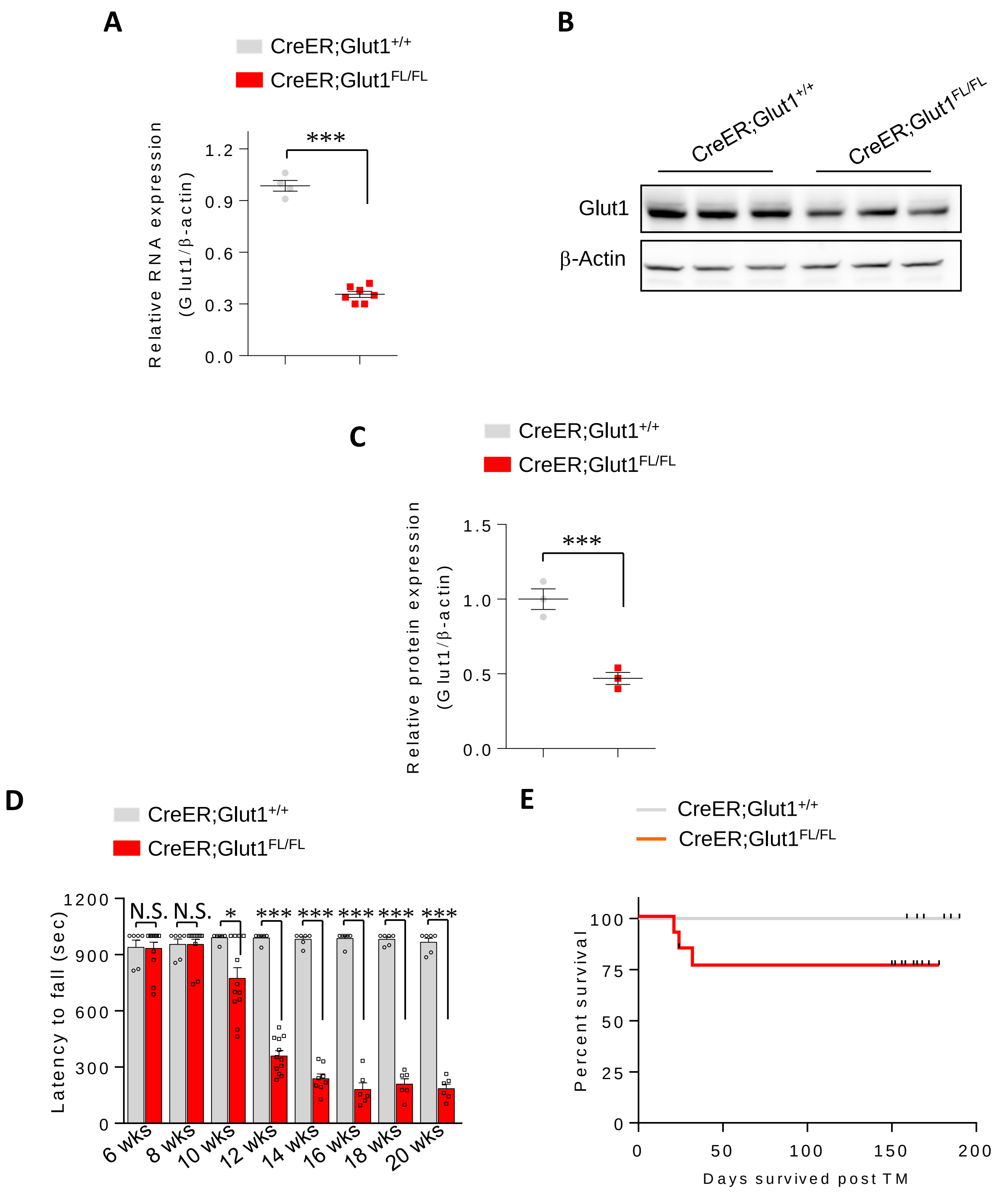

Supplementary Figure 9 – Depleting Glut1 in adult mice below heterozygous levels triggers disease. (A) Marked reduction in Glut1 transcripts in brain tissue of 5-month old *CreER;Glut1$^{FL/FL}$* mutants administered tamoxifen at 8 weeks of age to inactivate the floxed alleles; ***, $P < 0.001$, *t* test, n=3 controls and $n=7$ mutant mice. **(B)** Western blot of Glut1 protein in brain tissue of *CreER;Glut1$^{FL/FL}$* mutants treated with tamoxifen at 8 weeks of age. **(C)** Quantified result of Glut1 protein in brain tissue of tamoxifen-treated *CreER;Glut1$^{FL/FL}$* mutants; ***, $P < 0.001$, *t* test, n=3 mice of each cohort. **(D)** Depleting Glut1 below 50% in adult mice severely impairs motor performance on the rotarod. Note precipitous decline in performance 4 weeks after tamoxifen administration; *, ***, $P < 0.05$, $P < 0.001$ respectively, *t* test, $n \geq 6$ mice of each cohort analyzed. **(E)** Kaplan-Meier survival curves depict a modest reduction in lifespan of *CreER;Glut1$^{FL/FL}$* mutants depleted of Glut1 below heterozygous levels during adult life; n=10 mice of each cohort.

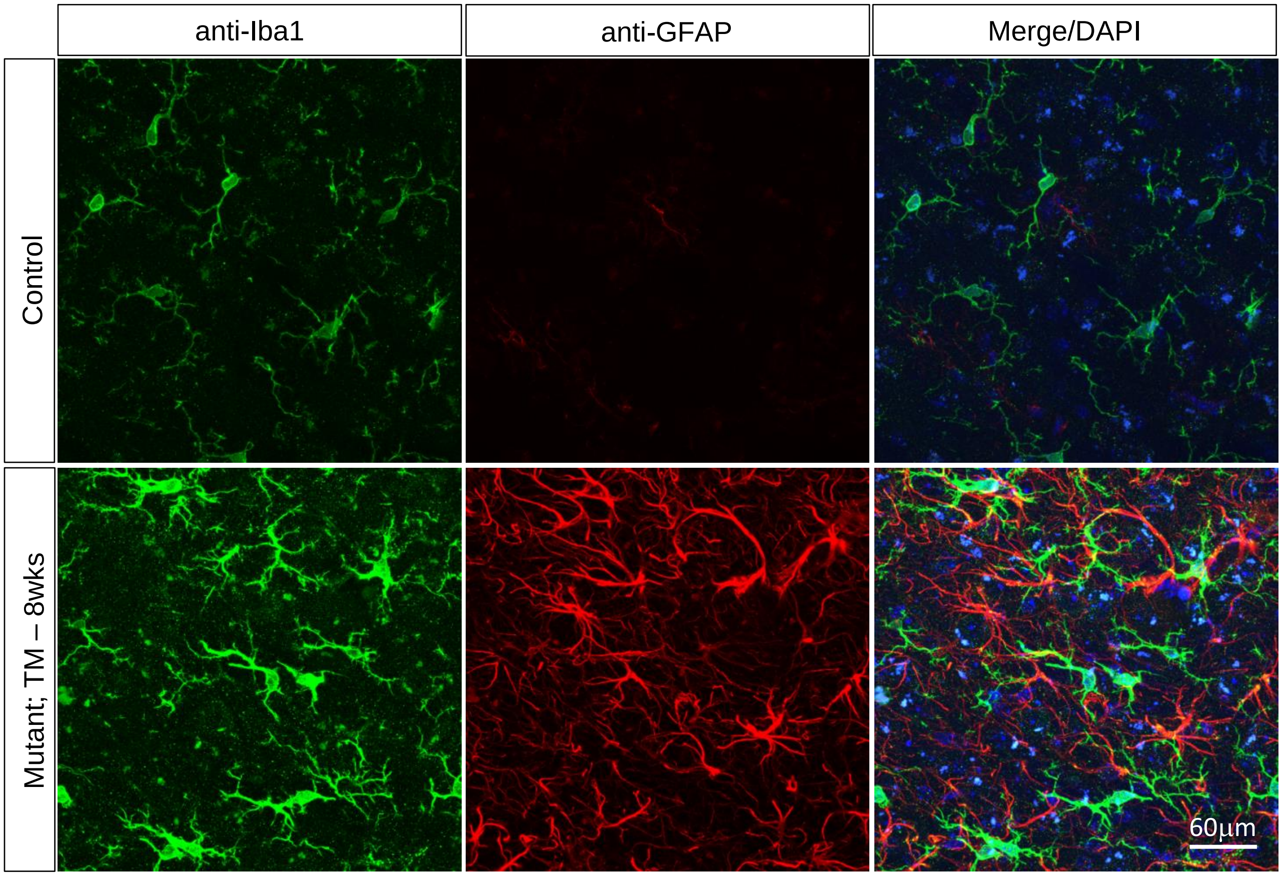

Supplementary Figure 10 – Late-onset gliosis of the brain in *CreER;Glut1$^{FL/+}$* mutants. Representative thalamic brain sections from 12-month old mutants depleted of Glut1 during adult life. Persistent Glut1 haplo-insufficiency eventually triggers a severe neuro-inflammatory response.

Supplementary Information

Rotarod test. To administer the rotarod test, mice were subjected to a training period of 5 minutes on an accelerating rotarod (Ugo Basile Inc., Italy) three times a day for four consecutive days. Measurements were recorded on the fifth day at a setting of 25rpm. Duration of time on the rotating rod was recorded and the experiment terminated if a mouse surpassed 1000s.

Brain parenchymal and vessel fractions. Mice were perfused with 1X PBS, whole brains extracted and the tissue gently homogenized by means of Dounce-type glass homogenizer in 1ml PBS. The extract was centrifuged at 1000g for 5min, the resulting pellet re-suspended once again in 1ml PBS and the centrifugation step repeated. The supernatant was then removed and the pellet re-suspended in 1ml of an 18% dextran solution in PBS. This suspension was centrifuged at 10,000g for 1min, the pellet saved and the supernatant containing the parenchymal fraction transferred to new tube. The pellet was once again re-suspended in 1ml of the 18% dextran and the centrifugation repeated. This process was repeated a third time and the pellets containing the vessel fractions were pooled and stored at -80°C until use. The supernatant fractions – containing the neuropil – were similarly combined and stored until use.

Quantification of neurons and activated glia in thalamic brain. Thalamic brain in the region of the ventral posteromedial (VPM) nucleus was imaged at a magnification of 10X and the external medullary lamina used as an anatomical marker to demarcate identical regions of the nucleus in the different mice. Neurons within the demarcated (also see fig. S7) dorsal two-thirds of the nucleus (dorsal VPM) were enumerated. Counts were conducted by the ImageJ software suite (NIH, Bethesda, MD). Activated glial cells were enumerated manually in images of the dorsal VPM acquired at a magnification of 63X.

Live-imaging of brain microvasculature. Five month old mice were anesthetized (1500 mg/kg urethane and 500 mg/kg glycopyrrolate, administered I.P.). Next, a craniotomy on the right hemisphere between bregma and lamda was performed and then fluorescein-conjugated dextran (2000 kDa, 0.1 ml from 25 mg/ml) injected into the tail vein to enable visualization of the capillaries. Images were acquired using a home-built two-photon laser scanning microscope (*41, 42*) equipped with a 20X, 0.95 NA objective lens (XLUMPLanFl,

Olympus). Stacks of angiograms (~ 510 x 510µm) were constructed beginning at the cortical surface down to a depth of ~ 500µm. Images were acquired every 2µm in the z-axis. Microvascular length was quantified by modifying an image processing pipeline previously described (*42*). Image analysis was performed using ImageJ and MATLAB. Briefly, three sub-regions (510 x 510 x 20µm) at depths of 200, 300 and 400µm were selected from the stack. The mean image of the sub-region was first pre-processed with a tubeness filter to enhance the features of the vessels. Then, an automatic intensity thresholding was applied to segment the vessels. Capillary diameter was further determined by skeletonization and Euclidean distance map. Only blood vessel segments with diameters of < 6µm were included in the final result.

Supplemental Information

PCR primers.

Primer	SOURCE	SEQUENCE
Primers for PCR		
Cre-ER^{T2} Mutant Rvs	IDT	5'-CGGTTATTCAACTTGCACCA-3'
Cre-ER^{T2} Common Fwd	IDT	5'-AAGGGAGCTGCAGTGGAGTA-3'
Cre-ER^{T2} Wild type Rvs	IDT	5'-CCGAAAATCTGTGGGAAGTC-3'
LoxP Fwd	IDT	5'-CTGTGAGTTCCTGAGACCCTG-3'
LoxP Rvs	IDT	5'-CCCAGGCAAGGAAGTAGTTC-3'
Glut-1 $^{\Delta/+}$ Fwd	IDT	5'-CTGTGAGTTCCTGAGACCCTG-3'
Glut-1 $^{\Delta/+}$ Rvs	IDT	5'-GAAGGCACATATGAAACAAATG-3'
Zp3 Cre transgene Fwd	IDT	5'-CGAGATTGAGGGAAGCAGAG-3'
Zp3 Cre transgene Rvs	IDT	5'-CAGGTTCTTGCGAACCTCAT-3'
Zp3 Cre Internal Positive Control Fwd	IDT	5'-AGTGGCCTCTTCCAGAAATG-3'
Zp3 Cre Internal Positive Control Rvs	IDT	5'-TGCGACTGTGTCTGATTTCC-3'
Tie2-Cre Fwd	IDT	5'-GGCAAATTTTGGTGTACGGTC-3'
Tie2-Cre Rvs	IDT	5'-CCTGTGCTCAGACAGAAATG-3'
Primers for qRT-PCR		
Glut-1 Fwd	IDT	5'-CTTGCTTGTAGAGTGACGATC-3'
Glut-1 Rvs	IDT	5'-CAGTGATCCGAGCACTGCTC-3'
BDNF Fwd	IDT	5'-TGGCCCTGCGGAGGCTAAGT-3'

BDNF Rvs	IDT	5'-AGGGTGCTTCCGAGCCTTCCT-3'
β-actin Fwd	IDT	5'-TGTTACCAACTGGGACGACA-3'
β-actin Rvs	IDT	5'-GGGGTGTTGAAGGTCTCAAA-3'
p53 Fwd	IDT	5'-GCCAAGTCTGTTATGTGCAC-3'
p53 Rvs	IDT	5'-GACTTCTTGTAGATGGCCATG-3'
p21 Fwd	IDT	5'- GACATTCAGAGCCACAGGCACC -3'
p21 Rvs	IDT	5'- GAGCGCATCGCAATCACGGCGC -3'
Ccng1 Fwd	IDT	5'-TTATGGGACGTAAGGAGACACC-3'
Ccng1 Rvs	IDT	5'-ATGGTTCCAGCTACTCTAGGTTG-3'
Gtse1 Fwd	IDT	5'-TGACAAAGAGAACGTGGACTCAC-3'
Gtse1 Rvs	IDT	5'-GAGGTGGGAGGCTTAGGTTC-3'
Perp Fwd	IDT	5'-TTTGGGAATGCGTGTCTCTG-3'
Perp Rvs	IDT	5'-TCAACTGTCTTTGCAGCACC-3'
Sesn1 Fwd	IDT	5'-TTCTCTCAGCCTGGAGGACAG-3'
Sesn1 Rvs	IDT	5'-CTTCAAAGTCAGGGTCCCGA-3'
GAPDH Fwd	IDT	5'-CGACTTCAACAGCAACTCCCACTCTTCC-3'
GAPDH Rvs	IDT	5'-TGGGTGGTCCAGGGTTTCTTACTCCTT-3'

Key reagents.

REAGENT or RESOURCE	SOURCE	IDENTIFIER
Antibodies for Western blotting		
Glut1 (1:5000)	Millipore	Cat#07-1401
Vinculin (1:2000)	Abcam	Cat#ab129002
β-actin (1:5000)	Sigma	Cat#A5441
Donkey anti-rabbit IgG (1:10,000)	Santa Cruz	Cat#sc-2313
Goat anti-mouse IgG (1:10,000)	Jackson Immunores.	Cat#115-035-003
phospho-AMPKα (Thr172) (1:1000)	Cell Signaling	Cat#2531
GAPDH	Santa Cruz Biotech.	Sc-32233
Antibodies for Immunostaining		
Lectin (1:1000)	Vector Laboratories	Cat#FL-1171
Glut1 (1:500)	Abcam	Cat#ab40084
GFAP (1:500)	Abcam	Cat#ab134436

GFAP (1:500)	Sigma	Cat#G3893
Iba-1 (1:500)	Wako	Cat#019-19741
CD11b (1:500)	BD Biosciences	Cat#553308
NeuN (1:500)	Synaptic Systems	Cat#266006
BDNF (1:500)	ABclonal	Cat# A1307
Alexa Fluor 488 goat α-rabbit (1:1000)	Abcam	Cat#ab150085
Alexa Fluor 488 goat α-mouse (1:1000)	Invitrogen	Cat#A11029
Alexa Fluor 568 goat α-rat (1:1000)	Invitrogen	Cat#A-11077
Alexa Fluor 568 donkey α-mouse (1:1000)	Invitrogen	Cat#A10037
Alexa Fluor 647 goat α-chicken (1:1000)	Abcam	Cat#ab150171
Experimental Models: Organisms/Strains		
Glut-1 $^{FL/FL}$ mouse	Young et al., 2011	N/A
Zp3-Cre mouse	Jackson Laboratory	Stock #003651
R26-CreERT2 mouse	Jackson Laboratory	Stock #008463
Tie2-Cre mouse	Jackson Laboratory	Stock #008863
Software and Algorithms		
GraphPad Prism	Graph Pad Software	https://www.graphpad.com/scientificsoftware/prism/
ImageJ	NIH	https://imagej.nih.gov/ij/
MATLAB	MathWorks	https://www.mathworks.com/products/matlab.html
ImageQuantTL	GE Healthcare	https://www.gelifesciences.com/
Leica LAS X	Leica	https://www.leica-microsystems.com/

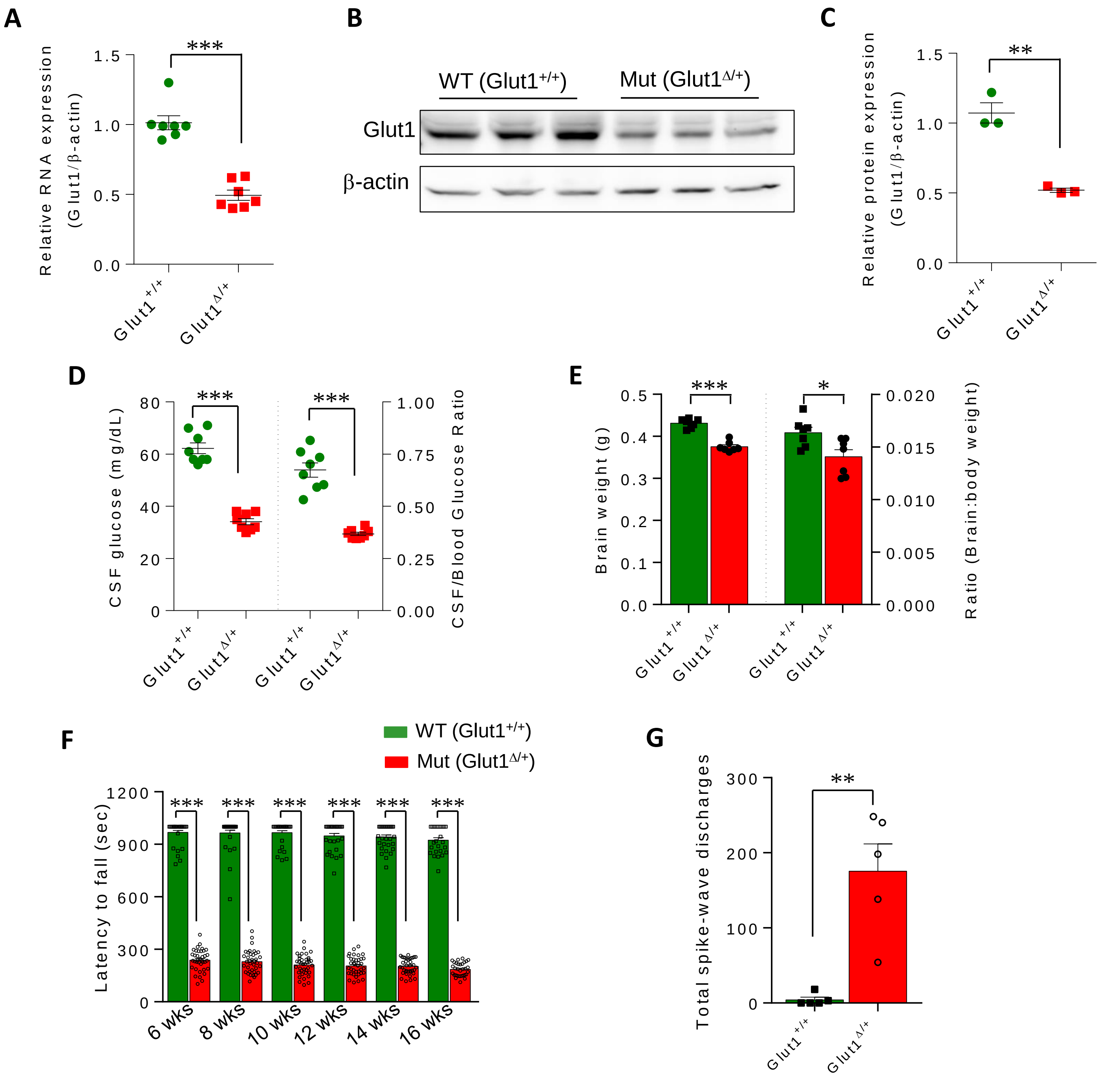

Supplementary Figure 1 - Inactivation of a floxed *Slc2a1 (Glut1)* allele evokes a classic Glut1 DS disease phenotype. (A) Graph depicts reduced Glut1 transcript in brain tissue of *Glut1*$^{\Delta/+}$ mutants. ***, $P < 0.001$, *t* test, *n*=7 mice of each cohort. **(B)** Western blot and **(C)** quantified result of blot depicting reduced Glut1 protein in brain of *Glut1*$^{\Delta/+}$ mutants. **, $P < 0.01$, *t* test, *n*=3 mice of each cohort. Evidence of **(D)** hypoglycorrhachia, **(E)** micrencephaly and **(F)** poor motor performance on the rotarod in *Glut1*$^{\Delta/+}$ mutants. **(G)** EEG abnormalities in the form of spike-wave discharges become evident in *Glut1*$^{\Delta/+}$ mutants. Note: *, **, ***, $P < 0.05$, $P < 0.01$, $P < 0.001$ respectively, *t* test; *n*=8 (panel D), *n*=7 (panel E), *n*≥30 (panel F) and *n*=5 (panel G).

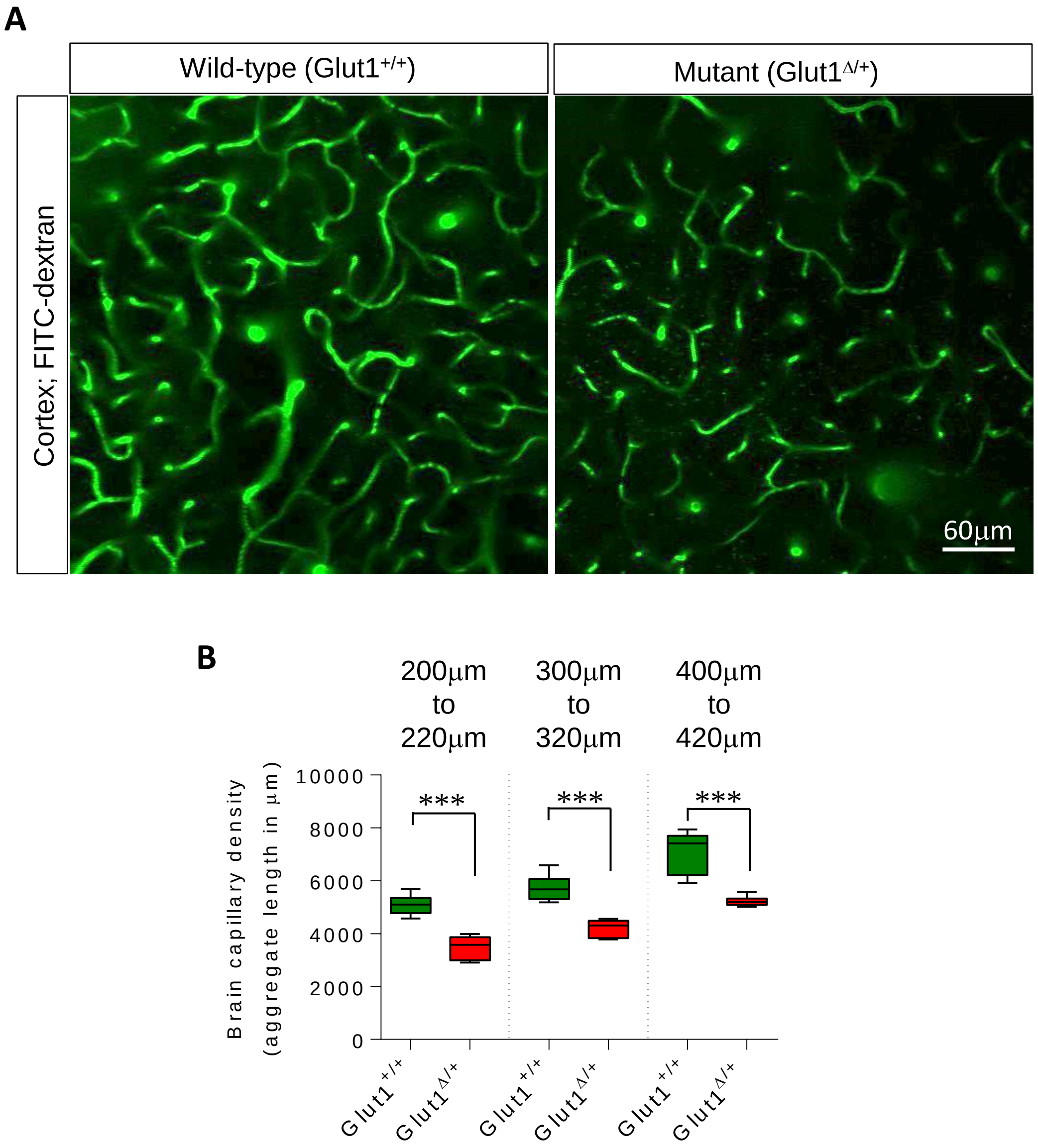

Supplementary Figure 2 – Fewer brain capillaries in *Glut1*$^{\Delta/+}$ mutant mice. (A) Representative live-imaging experimental result of the cortical brain microvasculature, at a depth of 200µm to 220µm, of a mutant and a control mouse. Note reduced density of capillaries in the mutant. **(B)** Graphical representation of cortical capillary densities at indicated depths in the two groups of mice following 2-photon live imaging; ***, $P < 0.001$, t test, $n \geq 9$ regions from each of N=6 mice of each cohort.

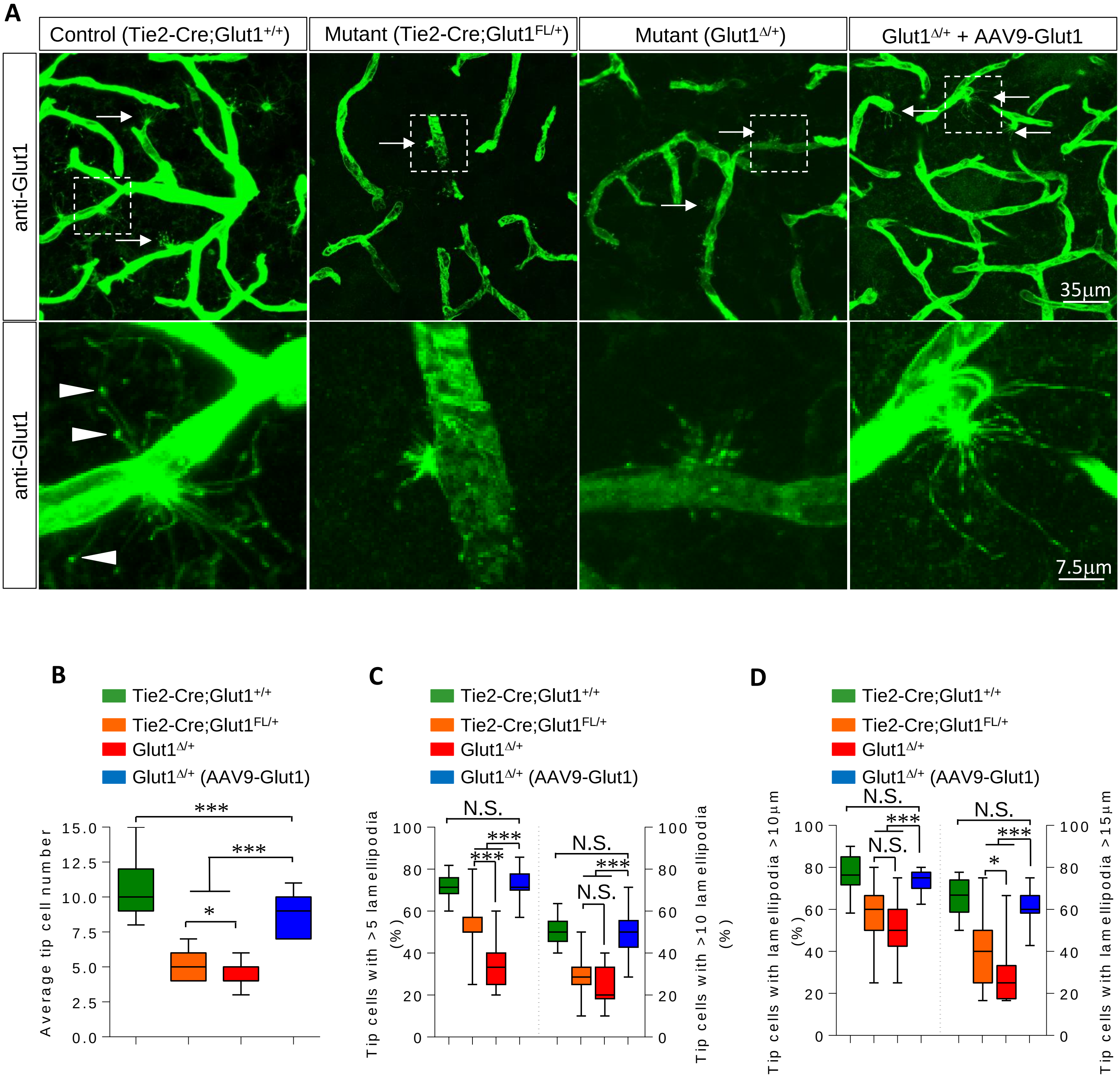

Supplementary Figure 3 – Endothelial tip cell pathology in constitutive and EC-specific Glut1 DS mutants. (A) Tip cell defects are evident in Glut1-stained cortical sections of PND14 *Glut1*$^{\Delta/+}$ and *Tie2-Cre;Glut1*$^{FL/+}$ mutants but rescued following repletion of the protein; tip cells indicated by arrows. Also depicted are magnified images of representative tip cells from each of the mice. Note the especially intense Glut1 staining at termini of control tip cell (arrowheads). Tip cell numbers are **(B)** reduced and have **(C)** significantly fewer and **(D)** shorter lamellipodia in *Glut1*$^{\Delta/+}$ and *Tie2-Cre;Glut1*$^{FL/+}$ mutants than they do in healthy controls or mutants restored for Glut1. Note: *,***, $P < 0.05$ and $P < 0.001$ respectively, *one-way ANOVA*, $n \geq 9$ regions from each of N=3 mice of each genotype examined for panels B – D.

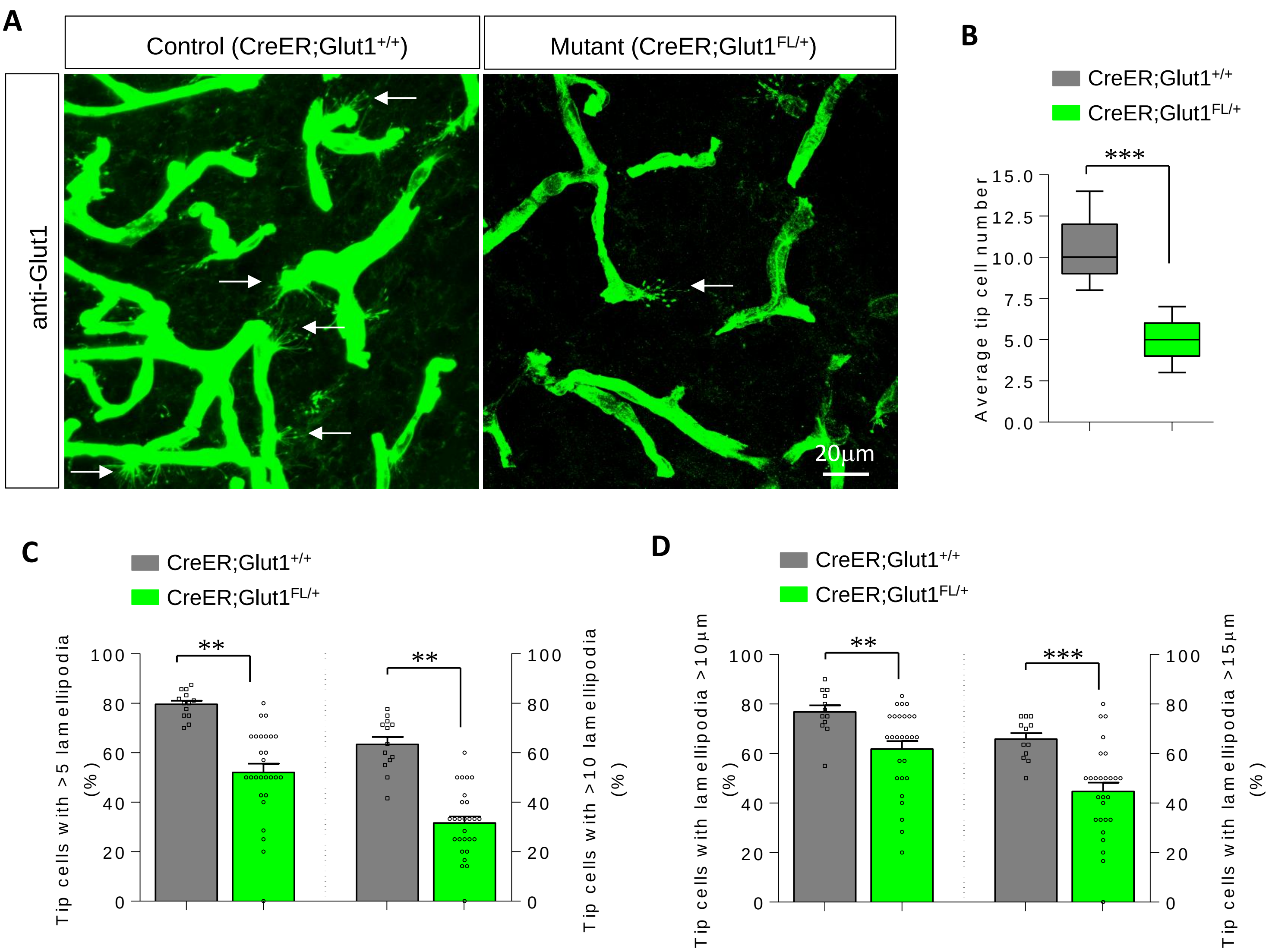

Supplementary Figure 4 – Endothelial tip cell defects in thalamic brain of mice depleted of Glut1 at PND2. (A) Thalamic brain sections of PND14 mice stained for Glut1 reveal a relative paucity and defects of mutant endothelial tip cells (arrows). Quantified results of **(B)** tip cell numbers, **(E)** lamellipodia counts and **(F)** lamellipodia size in mutants and controls. Note: **, ***, $P < 0.01$, $P < 0.001$ respectively, t test, $n \geq 9$ regions from each of N=3 mice of each genotype examined for panels B – D.

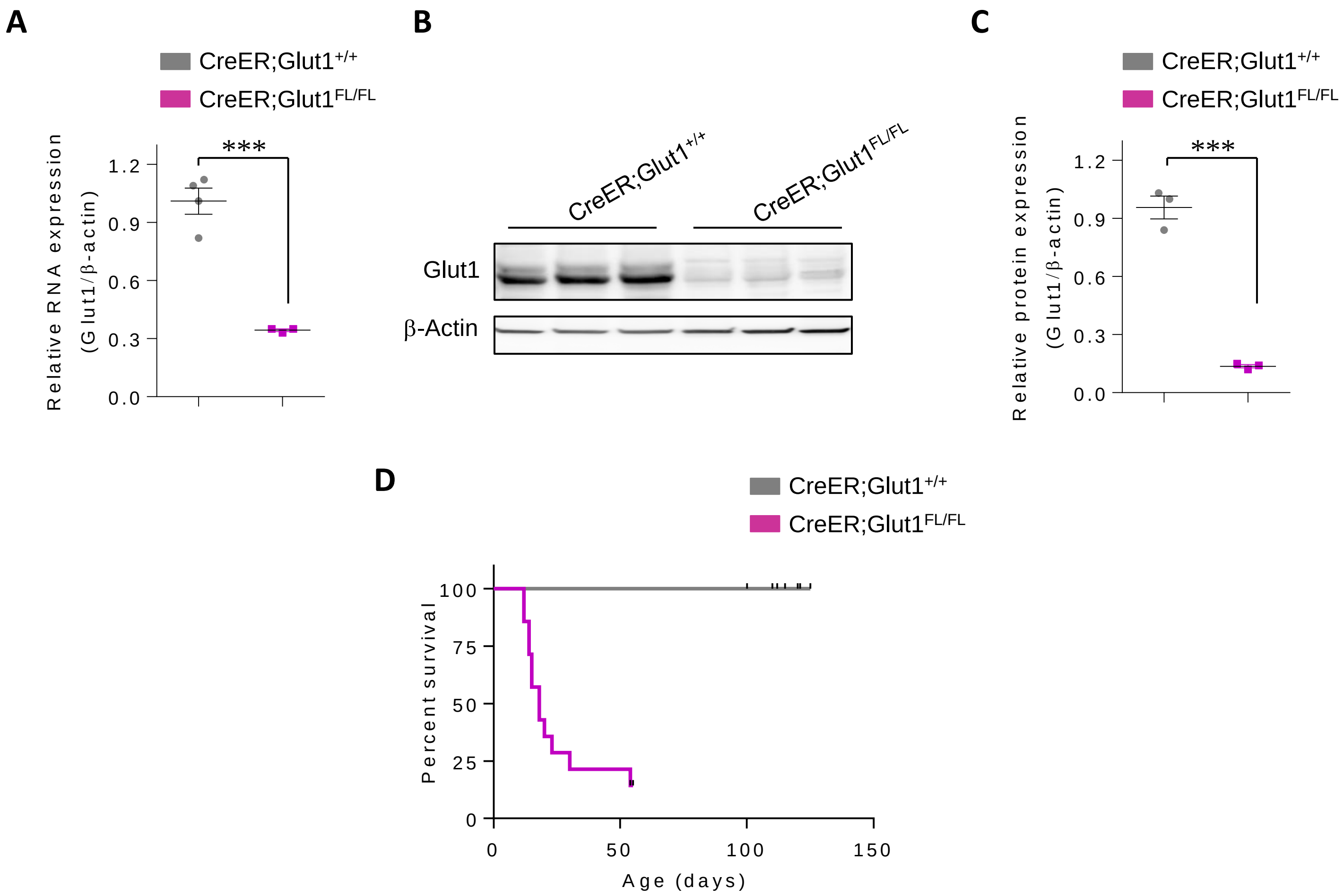

Supplementary Figure 5 – Early postnatal Glut1 depletion below heterozygous levels affects the viability of model mice. (A) Graph depicts reduced Glut1 transcript in brain tissue of treated *CreER;Glut1$^{FL/FL}$* mutants; ***, $P < 0.001$, *t* test, *n*=4 controls and 3 mutant mice analyzed. **(B)** Western blot and **(C)** quantified result of blot depicting reduced Glut1 protein in brain tissue of *CreER;Glut1$^{FL/FL}$* mutants; ***, $P < 0.001$, *t* test, *n*=3 mice analyzed. **(D)** Kaplan-Meier survival curves depict a marked reduction in lifespan of *CreER;Glut1$^{FL/FL}$* mutants depleted of Glut1 below heterozygous levels at PND2; $P < 0.0001$ between groups, Log-rank test, *n*=10 mice from each group.

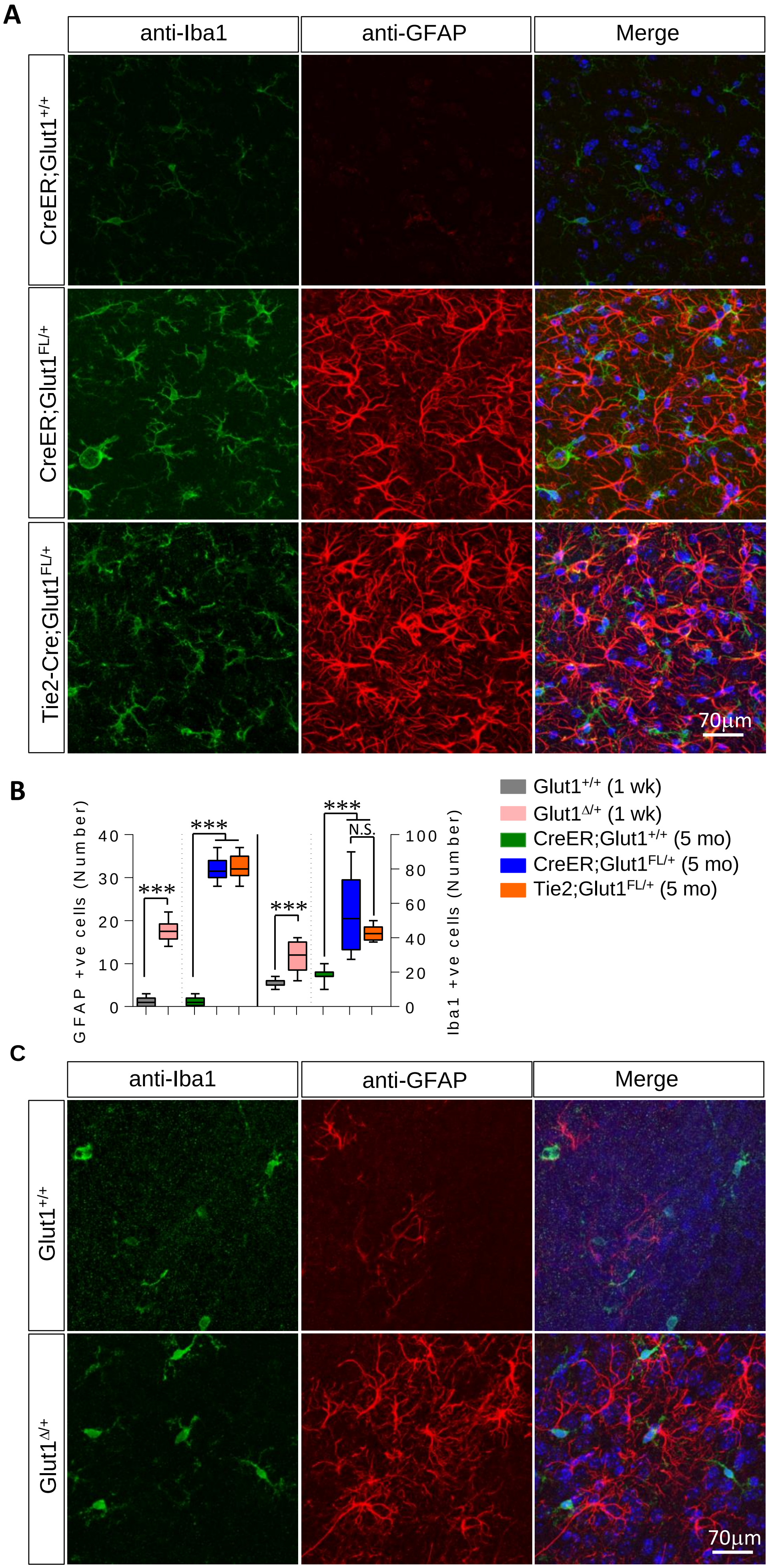

A
anti-Iba1
anti-GFAP
Merge
CreER;Glut1$^{+/+}$
CreER;Glut1$^{FL/+}$
Tie2-Cre;Glut1$^{FL/+}$
70µm
B
GFAP +ve cells (Number)
Iba1 +ve cells (Number)
0
10
20
30
40
0
20
40
60
80
100
***
***
***
***
N.S.
Glut1$^{+/+}$ (1 wk)
Glut1$^{\Delta/+}$ (1 wk)
CreER;Glut1$^{+/+}$ (5 mo)
CreER;Glut1$^{FL/+}$ (5 mo)
Tie2;Glut1$^{FL/+}$ (5 mo)
C
anti-Iba1
anti-GFAP
Merge
Glut1$^{+/+}$
Glut1$^{\Delta/+}$
70µm

Supplementary Figure 6 – Glut1 haploinsufficiency triggers early-onset brain neuro-inflammation in model mice. (A) Severe gliosis featuring activated microglia and reactive astrocytes in thalamic brain tissue (ventral posteromedial nucleus – VPM) of 5-month old mutants ubiquitously depleted of Glut1 at PND2 or selectively depleted of the protein in ECs during embryonic development. **(B)** Enumeration of reactive astrocytes and activated microglia; ***, $P < 0.001$, *t* test or one-way ANOVA, $n \geq 9$ thalamic regions from each of $N=3$ mice of each genotype assessed. **(C)** Neuro-inflammation was detected as early as 1 week of age in $Glut1^{\Delta/+}$ mutants.

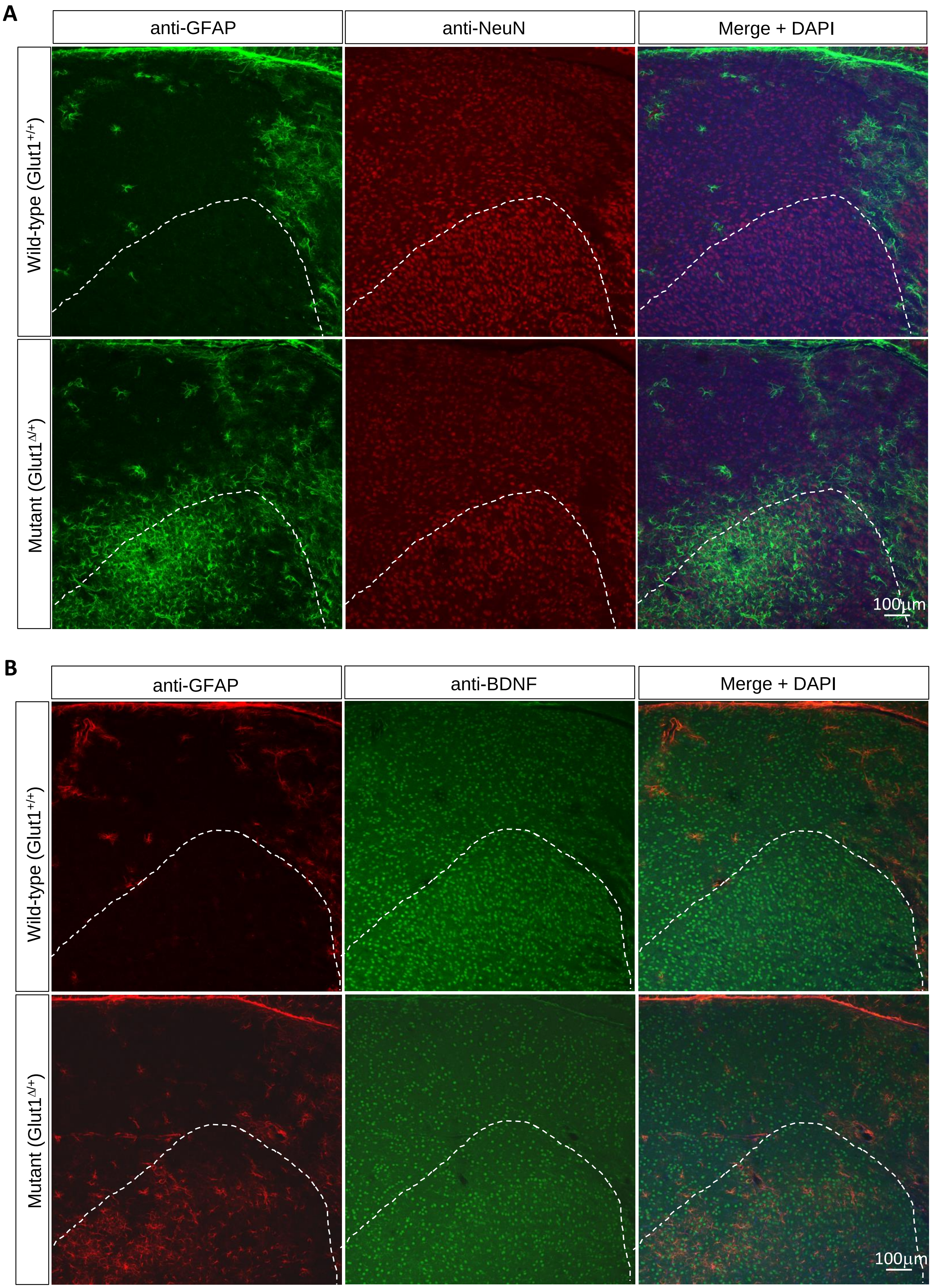
A
anti-GFAP
anti-NeuN
Merge + DAPI
Wild-type (Glut1$^{+/+}$)
Mutant (Glut1$^{\Delta/+}$)
100µm
B
anti-GFAP
anti-BDNF
Merge + DAPI
Wild-type (Glut1$^{+/+}$)
Mutant (Glut1$^{\Delta/+}$)
100µm

Supplementary Figure 7 – Glut1 DS is characterized by a deficiency of thalamic neurons. (A) Representative immuno-histochemically stained brain sections illustrate the correlation between the especially prominent astrocytosis of the mutant ventral posteromedial (VPM) thalamic nucleus (demarcated area) and the reduced numbers of neurons within this nucleus. **(B)** Sections from the same region of the brain sampled in panel (A) reveal fewer BDNF-positive cells in the mutant mouse.

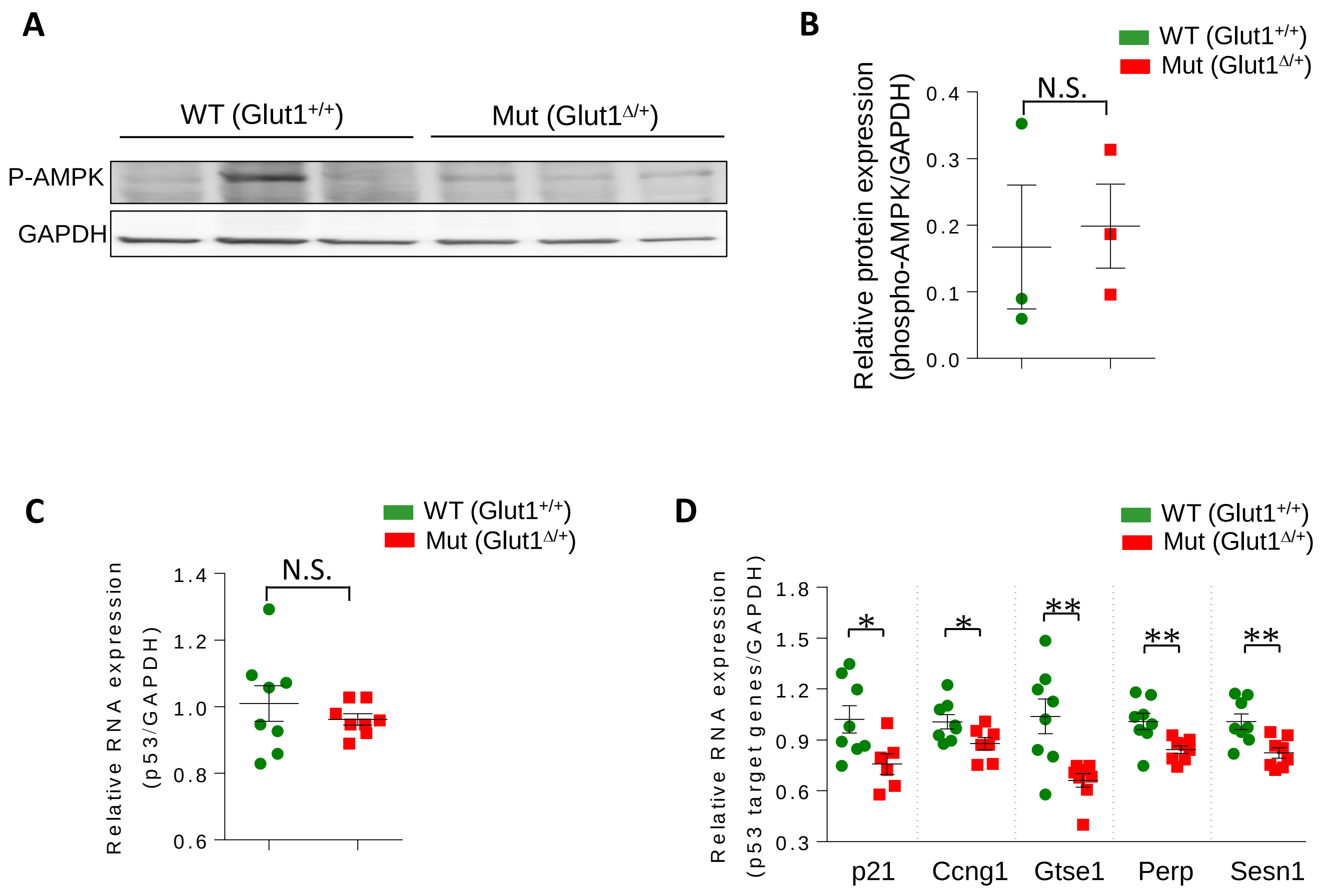

Supplementary Figure 8 – Glut1 haploinsufficiency does not alter AMPK levels or induce p53. (A) Western blot of phospho-AMPK levels in brain tissue from adult *Glut1*$^{\Delta/+}$ mutants and *Glut1*$^{+/+}$ controls. **(B)** Quantified results of western blot in panel A. Note: N.S. denotes $P > 0.05$, t tests, n=3 mice of each cohort. **(C)** p53 transcript levels remain unchanged in brain tissue of *Glut1*$^{\Delta/+}$ mutants. **(D)** RNA levels of p53 transcriptional targets are reduced in brain tissue of *Glut1*$^{\Delta/+}$ mutants. *, **, $P < 0.05$, $P < 0.01$, t tests, n=8 mice of each genotype assessed in panels C, D.

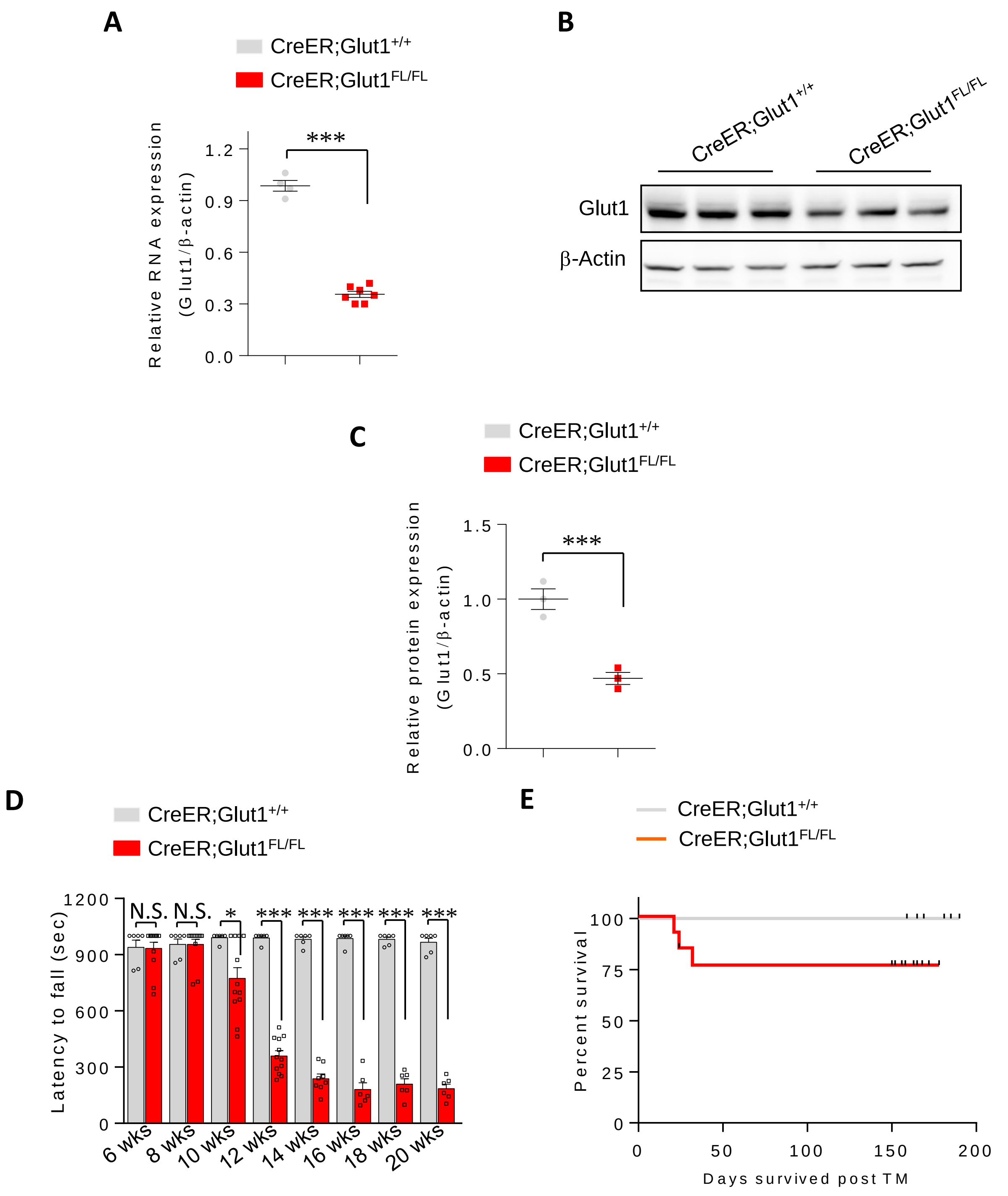

Supplementary Figure 9 – Depleting Glut1 in adult mice below heterozygous levels triggers disease. (A) Marked reduction in Glut1 transcripts in brain tissue of 5-month old *CreER;Glut1*$^{FL/FL}$ mutants administered tamoxifen at 8 weeks of age to inactivate the floxed alleles; ***, $P < 0.001$, *t* test, n=3 controls and n=7 mutant mice. **(B)** Western blot of Glut1 protein in brain tissue of *CreER;Glut1*$^{FL/FL}$ mutants treated with tamoxifen at 8 weeks of age. **(C)** Quantified result of Glut1 protein in brain tissue of tamoxifen-treated *CreER;Glut1*$^{FL/FL}$ mutants; ***, $P < 0.001$, *t* test, n=3 mice of each cohort. **(D)** Depleting Glut1 below 50% in adult mice severely impairs motor performance on the rotarod. Note precipitous decline in performance 4 weeks after tamoxifen administration; *, ***, $P < 0.05$, $P < 0.001$ respectively, *t* test, $n \geq 6$ mice of each cohort analyzed. **(E)** Kaplan-Meier survival curves depict a modest reduction in lifespan of *CreER;Glut1*$^{FL/FL}$ mutants depleted of Glut1 below heterozygous levels during adult life; n=10 mice of each cohort.

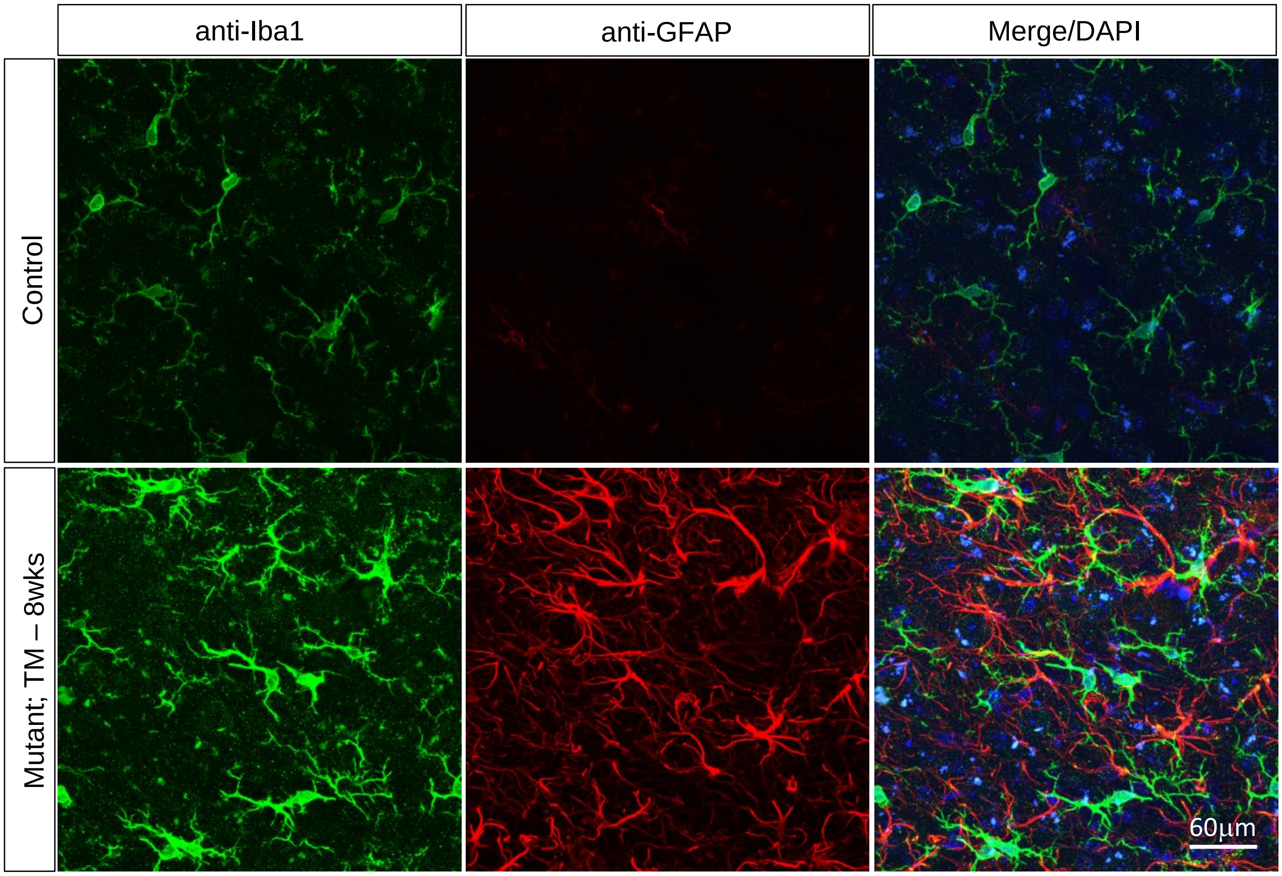

Supplementary Figure 10 – Late-onset gliosis of the brain in *CreER;Glut1$^{FL/+}$* mutants. Representative thalamic brain sections from 12-month old mutants depleted of Glut1 during adult life. Persistent Glut1 haplo-insufficiency eventually triggers a severe neuro-inflammatory response.
